# Use of a Web-Based Dietary Assessment Tool (RiksmatenFlex) in Swedish Adolescents: Comparison and Validation Study

**DOI:** 10.2196/12572

**Published:** 2019-10-04

**Authors:** Anna Karin Lindroos, Jessica Petrelius Sipinen, Cecilia Axelsson, Gisela Nyberg, Rikard Landberg, Per Leanderson, Marianne Arnemo, Eva Warensjö Lemming

**Affiliations:** 1 Swedish National Food Agency Uppsala Sweden; 2 Department of Internal Medicine and Clinical Nutrition Sahlgrenska Academy University of Gothenburg Gothenburg Sweden; 3 Department of Public Health Sciences Karolinska Institutet Stockholm Sweden; 4 Department of Biology and Biological Engineering Chalmers University of Technology Gothenburg Sweden; 5 Occupational and Environmental Medicine Center Department of Clinical and Experimental Medicine Linköping University Linköping Sweden

**Keywords:** dietary assessment, 24-hour hour dietary recalls, internet, validity, biomarkers, carotenoids, alkylresorcinols, adolescents

## Abstract

**Background:**

A Web-based dietary assessment tool—RiksmatenFlex—was developed for the national dietary survey of adolescents in Sweden.

**Objective:**

This study aimed to describe the Web-based method RiksmatenFlex and to test the validity of the reported dietary intake by comparing dietary intake with 24-hour dietary recalls (recall interviews), estimated energy expenditure, and biomarkers.

**Methods:**

Adolescents aged 11-12, 14-15, and 17-18 years were recruited through schools. In total, 78 students had complete dietary information and were included in the study. Diet was reported a few weeks apart with either RiksmatenFlexDiet (the day before and a random later day) or recall interviews (face-to-face, a random day later by phone) in a cross-over, randomized design. At a school visit, weight and height were measured and blood samples were drawn for biomarker analyses. Students wore an accelerometer for 7 days for physical activity measurements. Dietary intake captured by both dietary methods was compared, and energy intake captured by both methods was compared with the accelerometer-estimated energy expenditure (EEest). Intake of whole grain wheat and rye and fruit and vegetables by both methods was compared with alkylresorcinol and carotenoid concentrations in plasma, respectively.

**Results:**

The mean of the reported energy intake was 8.92 (SD 2.77) MJ by RiksmatenFlexDiet and 8.04 (SD 2.67) MJ by the recall interviews (*P*=.01). Intake of fruit and vegetables was 224 (169) g and 227 (150) g, and whole grain wheat and rye intake was 12.4 (SD 13.2) g and 12.0 (SD 13.1) g, respectively; the intakes of fruit and vegetables as well as whole grain wheat and rye did not differ between methods. Intraclass correlation coefficients ranged from 0.57 for protein and carbohydrates to 0.23 for vegetables. Energy intake by RiksmatenFlexDiet was overreported by 8% (*P*=.03) but not by the recall interviews (*P*=.53) compared with EEest. The Spearman correlation coefficient between reported energy intake and EEest was 0.34 (*P*=.008) for RiksmatenFlexDiet and 0.16 (*P*=.21) for the recall interviews. Spearman correlation coefficient between whole grain wheat and rye and plasma total alkylresorcinol homologs was 0.36 (*P*=.002) for RiksmatenFlexDiet and 0.29 (*P*=.02) for the recall interviews. Spearman correlations between intake of fruit and vegetables and plasma carotenoids were weak for both dietary tools. The strongest correlations were observed between fruit and vegetable intake and lutein/zeaxanthin for RiksmatenFlexDiet (0.46; *P*<.001) and for recall interviews (0.28; *P*=.02).

**Conclusions:**

RiksmatenFlexDiet provides information on energy, fruit, vegetables, and whole grain wheat and rye intake, which is comparable with intake obtained from recall interviews in Swedish adolescents. The results are promising for cost-effective dietary data collection in upcoming national dietary surveys and other studies in Sweden. Future research should focus on how, and if, new technological solutions could reduce dietary reporting biases.

## Introduction

There is a need for cost-effective dietary assessment methods that provide data of high quality to enable studies on diet and health. Open-ended dietary assessment methods such as food diaries and interviewer-administered 24-hour dietary recalls are resource demanding. Food Frequency Questionnaires (FFQ) are therefore commonly used in large epidemiological studies to minimize costs and workload. FFQs, however, are less accurate than open-ended dietary assessment methods [1,2]. Furthermore, they provide less detail on the type and amounts of foods eaten and no information on meal patterns. Food consumption data need to be harmonized for dietary exposure assessments on a European level. To achieve harmonization, the European Food Safety Authority (EFSA) recommends two 24-hour dietary recall interviews with at least one face-to-face interview for national dietary surveys of adults and children from the age of 10 years [3].

In recent years, technological developments have made it possible to assess diet using online tools. This offers potential for cost savings in data collection [4], but biases related to the self-reported dietary intake may still be present [5]. Several computer and Web-based 24-hour dietary recall and food diary methods have been developed. In a recent review, 21 different 24-hour dietary recall tools for use in children and adults were identified [6]. Most of these tools were compared with 24-hour dietary recall interviews or food diaries, with generally good agreement between the methods [6]. Only a few Web-based 24-hour dietary recalls or food diaries have been validated against recovery biomarkers [7-11]. Several Web-based 24-hour dietary recall and food diary tools have been specifically developed or adapted for use in children and adolescents. These tools have been evaluated by comparison with 24-hour dietary recall interviews [12-16], food records [17], direct observation [12,14,18,19], estimated total energy expenditure based on accelerometer measurements [20,21], or concentration biomarkers [19,22,23].

Dietary assessment methods designed to characterize usual intake are difficult to validate, as their actual validity cannot be estimated with absolute certainty [24]. Ideally, dietary assessment methods should be validated against recovery biomarkers to allow assessment of their measurement errors and calibration. However, only a few recovery biomarkers are available. The doubly labeled water method (measuring energy expenditure) for validating energy intake is expensive, and measurement of nitrogen in 24-hour urine collections for protein intake requires very motivated study participants. An alternative option to validate energy intake is estimation of the total energy expenditure from accelerometer data and equations [20,21,25,26]. Although this estimation of energy expenditure is not without errors, the physical activity level is objectively measured. Thus, it can be assumed that errors are uncorrelated to subjectively reported energy intake. Another option is to use concentration biomarkers to evaluate intake of specific food groups. Concentration biomarkers do not capture total intake, but can be used to evaluate the intake of specific food groups, although the strength of agreement is expected to be lower than that for recovery biomarkers [27]. A high intake of whole grains, fruits, and vegetables is associated with a decreased risk of chronic disease [28], and it is therefore important to be able to measure the intake of these food groups correctly. Alkylresorcinols are found in the outer parts of the wheat and rye kernels and can be used as biomarkers for whole grain wheat and rye intake [29-31]. Carotenoids reflect intake of fruit and vegetables satisfactorily and have been used in several studies to validate fruit and vegetable intake [32,33].

When planning the national dietary survey of adolescents in Sweden, there was an urgent need for a cost-effective, user-friendly dietary assessment method that could capture dietary intake satisfactorily and comply with EFSA’s guidelines [3]. Therefore, a new Web-based dietary assessment tool—RiksmatenFlex—was developed for the survey of adolescents. The intention was also to provide a Web-based tool that could be further adapted to other age groups and study populations. As pointed out by Eldridge et al [4], new technology tools for assessing dietary intake require detailed publications describing the tool and testing the validity in order to meet general quality standards. Thus, the overall aim of this study is to describe the RiksmatenFlex tool and evaluate the validity of the dietary registration part of the tool in adolescents. The validity was evaluated by (1) comparing the reported energy and macronutrient intake with interview-administered 24-hour dietary recalls; (2) comparing the reported energy intake with total energy expenditure estimated from weight, height, and accelerometer data; (3) comparing the intake of whole grain wheat and rye, fruits, and vegetables with the plasma concentration of biomarkers alkylresorcinols and carotenoids, respectively; and (4) evaluating performance of the tool in (2) and (3) with the performance of interviewer-administered 24-hour dietary recalls.

## Methods

### The RiksmatenFlex Tool

**Figure 1:**
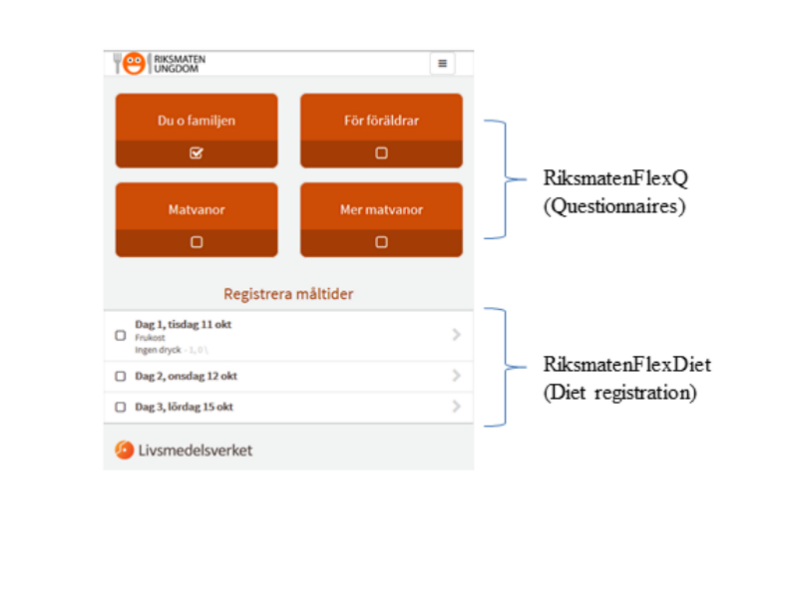
The start page of RiksmatenFlex.

The RiksmatenFlex is a self-administered, Web-based method for use on smartphones, tablets, and computers. It includes a diet registration part (RiksmatenFlexDiet) and a questionnaire part (RiksmatenFlexQ; Figure 1).

RiksmatenFlex was developed in 2014-2015 by the National Food Agency in Sweden (NFA) for the national dietary survey of adolescents. To ensure a user-friendly and attractive system, an expert in interaction design was also involved in the development in addition to experts in nutrition and information technology. The development was based on experiences from a Web-based food diary [8,34,35] and an interview study with adolescents (data not published). The process also included focus groups throughout the development and test sessions with adolescents. The system was Windows-based, and in 2015, the following development environment was used: SQL server 2014, Windows Server 2012, MVC 5, .NET 4 and .NET 4.5, and VS2013-17. The system is compatible with IE 9-11, Chrome, Win 7, and Office 2010 or later versions.

The Web page is accessed by individual usernames and passwords. At the first log in, participants provide their email and phone number for future communication. At this time, the dates for the registration days are also generated. RiksmatenFlexQ is described in more detail elsewhere [36].

### RiksmatenFlexDiet

#### Registration of Food and Drinks

**Figure 2:**
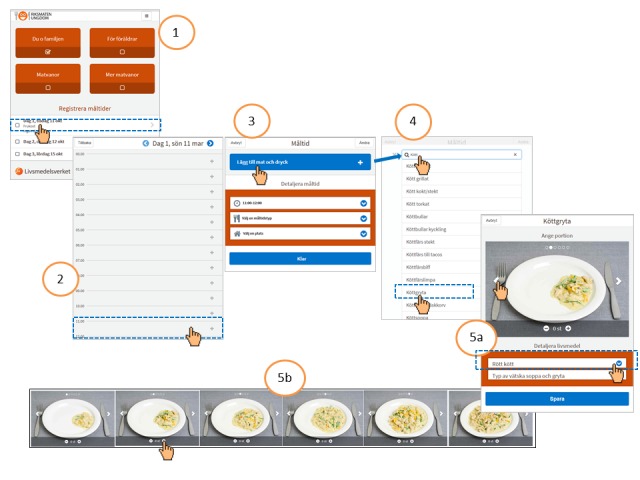
Overview of the different dietary recording steps in RiksmatenFlexDiet.

The dietary registration is based on the 24-hour dietary recall method. However, the method is flexible and can be used as a food diary. It can also be easily adapted for new studies and age groups other than adolescents. The different steps of RiksmatenFlexDiet are displayed in Swedish in Figure 2. In brief, the steps include (1) selection of the required recording day; (2) selection of the time of the eating/drinking occasion; (3) selection of the type of meal (breakfast, lunch, dinner/supper, snack, other eating, or drink only) and place of the eating/drinking occasion (at home, at school, in a restaurant/bar/café, at an event such as cinema/theatre/sports, someone else’s home, fast food restaurant, other place, on the way car/bus/train, or at work); (4) search for foods and drinks using a built-in search engine linked to a food list (see below); and (5) selection of portion size and, where relevant, specification of the details of the dish (for example, type of meat in a casserole).

To aid participants in finding the correct food, different spelling options and brand names were included in the search engine. Furthermore, pictures of foods commonly consumed in the five food categories—bread, ready-to-eat sandwiches, breakfast cereals, ice cream, and fat spreads—are linked to the search engine (Multimedia Appendix 1). The program uses machine learning to adapt the search list to user preferences.

RiksmatenFlexDiet is self-instructive and includes several logical reminders, for example, a reminder to help participants remember to register drinks. Further, before submitting the completed registration, participants are presented with a list of commonly forgotten foods and are asked to review all recorded foods and drinks. Participants are also asked to specify if the recording day was an ordinary day, not an ordinary day, or an ill day. Automatic reminders were sent to participants if a registration day was not completed. Time to complete one day’s tasks was 15-30 minutes, depending on age. All recorded foods and drinks, together with portion sizes, are directly stored in a database and automatic calculation of energy, nutrient, and food group intakes is enabled through direct link to the food composition database. The data output is flexible, and it is possible to extract foods from composite dishes and raw agricultural commodities or obtain intake by day, meal type, meal place, and time of the intake. All foods are FoodEx2 coded.

#### The Food List

**Figure 3:**
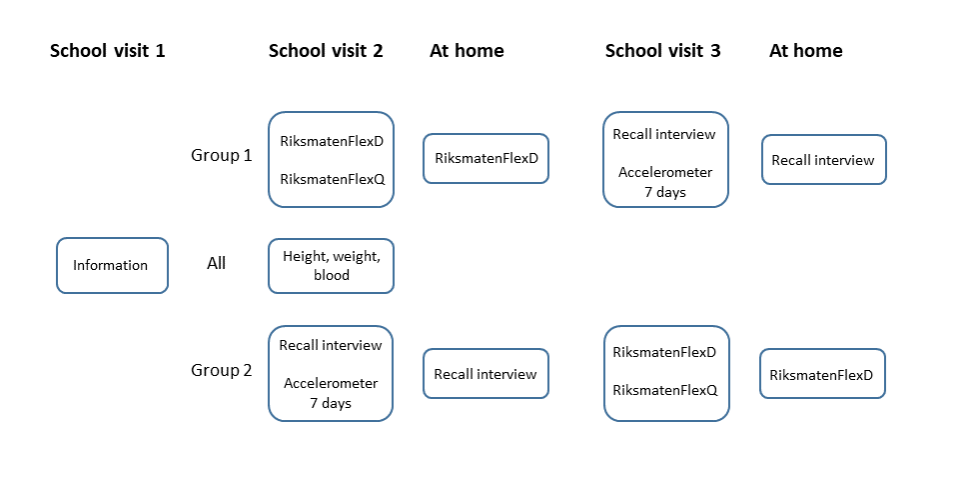
Study design of the validation study of RiksmatenFlexDiet. RiksmatenFlexD: RiksmatenFlexDiet.

**Figure 4:**
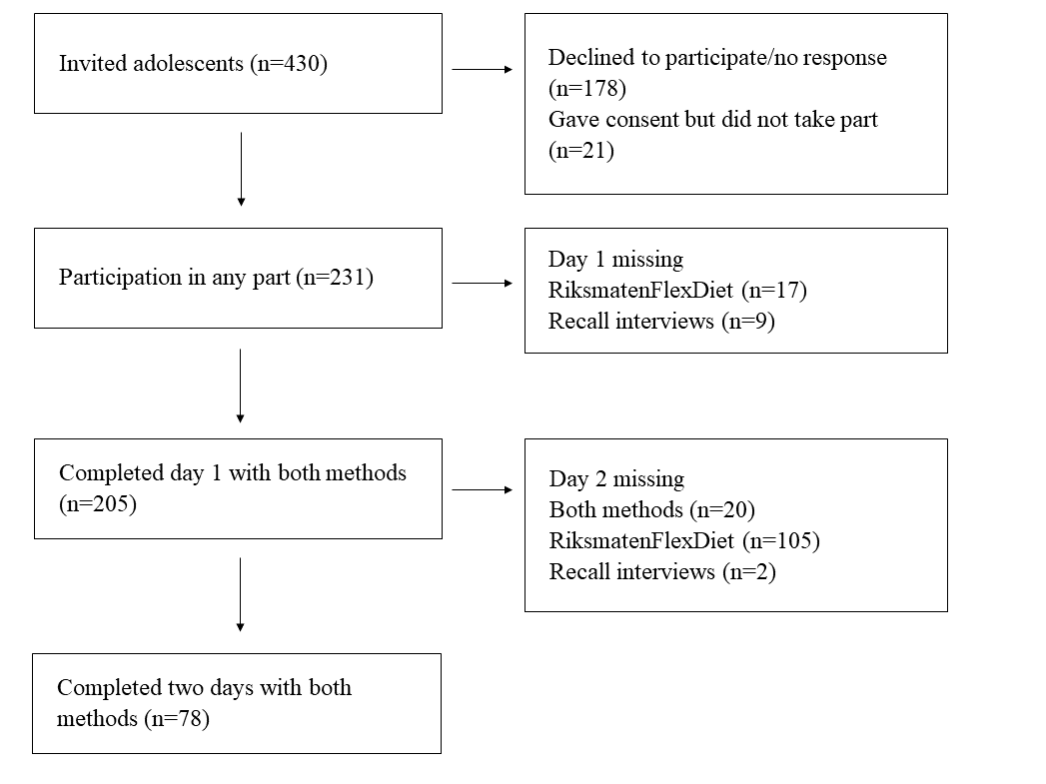
Participant flow of the validation study of RiksmatenFlexDiet.

**Figure 5:**
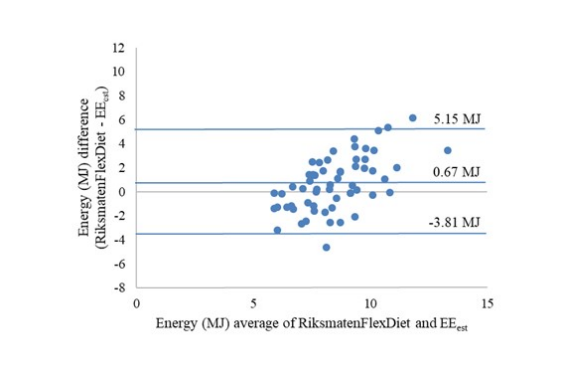
Bland-Altman plot of reported energy intake by RiksmatenFlexDiet and estimated energy expenditure (n=60). EEest: estimated total energy expenditure.

The food list compiled for RiksmatenFlexDiet is built on the Swedish national food composition database with the aim to represent Swedish adolescents’ food consumption. Foods were selected based on the amounts and frequencies of consumed foods, drinks, and dishes reported in a previous survey of adults [37] and a study of children aged 11-12 years (data not published). Key foods were also identified in focus groups with adolescents. To facilitate the search in RiksmatenFlexDiet, the number of foods was limited to approximately 800. Thus, some of the foods are generic, and sales statistics from commercial companies were used to compile the composition of these foods. For many generic foods, it is also possible to specify details. For example, the type of meat (pork, beef, etc) and liquid base (tomato, cream, etc) can be specified for a meat casserole (Figure 2-5a). However, brand names are not included in the specifications. This validation study included 761 core foods, but with the additional details, there were approximately 2300 possible food item combinations available. The food list is presented in Multimedia Appendix 2.

#### Picture Portion Guide

The picture portion guide included in RiksmatenFlexDiet is an extended version of the portion guide developed for the national dietary survey Riksmaten 2010-2011 [37]. The guide includes household measures, pieces, portion pictures of glasses and cups, and photos of 39 different food categories, with four to eight different reference sizes in each category Figure 2-5b. Six reference sizes were most common.

### The Validation and Comparison Study

#### Study Population

The study population consists of schoolchildren taking part in a feasibility study preceding the national dietary survey Riksmaten Adolescents 2016-2017.

We aimed for a sample size of 75 adolescents, based on the assumption that more than 50 participants are sufficient when validating dietary intake against biomarkers [38]. Participants were recruited from elementary and high schools in grades 5, 8, and 11 across Sweden. Schools were selected from the Swedish school unit register with the aim to recruit one class from each school grade and Occupational and Environmental Medicine Centre (OEMC) region. In total, 86 schools were contacted, 15 accepted participation, and 18 classes (one class in each grade and region) were selected to participate. All students in the 18 selected classes were invited to participate in the study. Exclusion criterion was not being able to read and write Swedish. For participants to be included in the validation analyses, they needed to have completed 2 days of diet registration/recall with both methods.

When a school agreed to take part in the study, information letters including consent forms were sent to the eligible participants as well as the parents of the students in grades 5 and 8 of the selected class. All participants consented to take part, and for children younger than 16 years, their legal guardians provided written consent. Participants were given a gift voucher of 300 Sk (Swedish krona). The Regional Ethical Review Board in Uppsala, Sweden, approved the study (2015/190).

#### Study Design

The RiksmatenFlexDiet was compared with 24-hour dietary recall interviews (recall interviews), and diet by both methods was compared with estimated energy expenditure and biomarkers. As interviews about food intake might influence how well the diet is later reported in the self-reported RiksmatenFlexDiet and because blood was only drawn at school visit 2, the participants were randomized to either start with RiksmatenFlexDiet or recall interviews. The randomization was performed at the individual level for one class at a time using a simple randomization procedure. Figure 3 provides an outline of the study design. The study started with an information session at school with the selected classes (visit 1) when the study staff informed the students about the study, and the students had the possibility to ask questions. At the first examination day (visit 2), the signed consent forms were collected by the study staff and the participants started with either RiksmatenFlexDiet or a recall interview. At visit 3, the participants changed to the other method. Time between school visits 2 and 3 was 2-4 weeks. For both methods, diet was reported on a random day at home, 1-2 weeks after the school visits. Weight and height were measured and blood was drawn at school visit 2. Trained research assistants recruited the classes, informed the schools and teachers about the study, and carried out the fieldwork. Phlebotomists from six of the seven OEMCs in Sweden carried out the blood sampling.

#### RiksmatenFlexDiet

RiksmatenFlexDiet was used both as a self-administered 24-hour dietary recall and as a food diary depending on day and age. The 24-hour dietary recall approach was used for the first day for all participants and for the second day for participants in grade 11. For the younger age groups (grade 5 and grade 8), the second day was recorded prospectively, as the interview study preceding the development of RiksmatenFlexDiet had indicated that they might have difficulties remembering yesterday’s intake. Day 1 intake was recorded at school and day 2 intake was recorded at home and randomly assigned to occur 7-14 days later. The first-day recording usually occurred during the school week, and around 80% of the second-day recordings were generated as a weekend day including Fridays. The date was unknown until the participants received an automated email with instructions to register food intake. Automatic email reminders were sent to participants if they did not complete their registrations. All records with an energy intake less than 800 kcal or over 3500 kcal were manually checked. Records with an intake below 800 kcal were judged as incomplete and excluded if no illness, less than three intake occasions (defined as at least one energy-contributing food item), and no meal after 3 PM was recorded (three records identified, one excluded). Records with an intake of over 3500 kcal were checked for obvious misunderstandings of amounts and foods (three records identified, none excluded).

The intake of energy, macronutrients, beta-carotene, and total whole grains was calculated using the NFA food composition database (the Riksmaten adolescent pilot study; the foods are displayed in Multimedia Appendix 2). Whole grain wheat and rye intake was calculated by excluding whole grains from sources other than wheat and rye in the total whole grain intake. Fruit and vegetable intake included all fruit (including berries) and vegetables (not potatoes and pulses) consumed, including both amounts directly recorded and amounts extracted from dishes. Juice was excluded from the intake due to the different nutrient density.

#### Recall Interviews

On day 1, a face-to-face interview was conducted in school and on day 2, the interview was performed over the phone. The second day was randomly assigned 7-14 days after the first interview, taking the weekday/weekend day into account as was done for RiksmatenFlexDiet. After the first interview, an appointment for the second phone interview was made with the participant. Trained staff with a nutritional background carried out all interviews, and the interviews followed a 24-hour dietary recall multiple pass protocol. The interview covered the period between waking up on the preceding day and waking up on the interview day. Household measures and a Web-based portion guide based on the pictures included in RiksmatenFlexDiet were used to describe the amounts of foods consumed. Participants were also asked if the previous day was a normal day. After the interview, the reported intake was coded using the nutrient calculation program Dietist Net Pro (Kost och Näringsdata AB, Stockholm, Sweden) and the NFA food composition database (recall interviews). Intake of whole grain wheat and rye, fruits, and vegetables was calculated as was done for RiksmatenFlexDiet.

#### Background Information

Educational levels of both parents were reported in RiksmatenFlexQ [36]. The information was combined and dichotomized to at least one parent with postsecondary education or no parent with postsecondary education.

#### Anthropometric Measurements and Blood Sampling

Height and weight were measured and nonfasting blood was collected from all participants at visit 2. Height was measured to the nearest 0.1 cm without shoes and weight, to the nearest 0.1 kg in light indoor clothing. Body mass index was calculated, and overweight/obesity was defined according to the recommendations by the International Obesity Task Force [39]. Height and weight were also used to estimate energy expenditure (see below).

#### Estimated Energy Expenditure

Physical activity was measured with an accelerometer (ActiGraph GT3X or wGT3X+, Tri-axis Accelerometer Monitor; Actigraph LLC, Pensacola, Florida). The participants were given the accelerometer after the first recall interview and instructed to wear it from waking up in the morning until bedtime for 7 consecutive days. The accelerometer was attached to an elastic band and placed on the right hip. Participants were instructed to remove the accelerometer when showering or doing water activities. Data were analyzed using ActiLife 6 Data Analysis Software (Actigraph LLC). Measurements of at least 3 complete days, including one weekend day (Saturday and Sunday), were considered as complete. Physical activity was assessed between 7 AM and 11 PM. The epoch length was set to 5 seconds. A complete day includes ˃500 minutes of registration. Based on mean counts per minute (cpm), physical activity energy expenditure was estimated using the modified prediction equations by Ekelund et al [40]:

Physical activity energy expenditure (kJ/day)= 4.182×(66.847+(cpm×0.953)–(176.91×sex)

Total energy expenditure was calculated as physical activity energy expenditure+basal metabolic rate+diet-induced thermogenesis, where diet-induced thermogenesis was estimated as 10% of the total energy expenditure [41]. The basal metabolic rate was estimated from the Henry equations based on age, sex, height, and weight [42].

#### Biochemical Analyses

Alkylresorcinols and carotenoids were analyzed in plasma. Five participants did not provide enough blood for the analyses. In addition, one alkylresorcinol analysis failed.

Gas chromatography-mass spectrometry (GC-MS) was used to quantify the alkylresorcinols C17:0-C25:0 in 0.2 mL plasma samples, as described previously [43]. The intra- and interassay coefficients of variation were both 15% for total alkylresorcinols.

Plasma concentrations of carotenoids were determined with high-performance liquid chromatography, as previously described [34]. The intra- and interassay coefficients of variation varied between 5% and 7%.

### Statistical Analysis

STATA statistical software (Version 14.2; STATA Corp, College Station, Texas) was used for the statistical analyses. Background information is presented as proportions and medians with the first and third quartiles. A mean of data from 2 days was used for all the analyses of reported dietary intake, and descriptive data are presented as mean and SD. Paired *t* test was used to test the difference in intake between the methods. Bland-Altman plots were used to assess precision and bias between reported intake by both methods and between reported energy intake by the respective method and estimated energy expenditure. Agreement for reported energy and macronutrient intake by the two methods was calculated by tertiles, and a linear weighted kappa was used to evaluate the agreement between classifications. Intraclass correlations between the two dietary methods were calculated using a two-way mixed-effects model to evaluate the strength of agreement. Energy-adjusted (g/MJ) intake of fruit and vegetables and whole grain wheat and rye were correlated with the respective biomarkers by calculating the Spearman rank correlation coefficients.

## Results

### Study Population and Reported Days

The participant flow is illustrated in Figure 4. A total of 430 adolescents were invited to participate, of which 397 were randomized to either start with RiksmatenFlexDiet (n=194) or recall interviews (n=203). In total, 231 (54%) participated in at least one part of the study, 205 participants completed the first day by both methods (113 started with RiksmatenFlexDiet and 118 started with recall interviews), and 78 participants completed 2 days by both methods (36 started with RiksmatenFlexDiet and 42 started with recall interviews). Eighteen of the participants with complete diet information did not have complete information from the accelerometer measurements.

Characteristics and plasma biomarkers of the 78 participants with complete diet information are presented in Table 1. Girls were more likely to participate than boys. The 60 participants with complete diet and accelerometer data were similar to the 78 participants with complete diet information. Lycopene was the most abundant carotenoid (36%), and the ratio of alkylresorcinol C17:0/C21:0 was 0.16.

The number of weekdays and weekend days (Friday-Sunday) was 93 (60%) and 63 (40%), respectively, for RiksmatenFlexDiet and 96 (62%) and 60 (38%), respectively, for recall interviews, which was close to the expected distribution for both methods.

**Table 1:** Characteristics, plasma concentrations, and counts per minute for participants with complete diet information (n=78).

Variables		Values
**Sex, n (%)**		
	Girls	54 (69)
	Boys	24 (31)
**Academic school year (years), n (%)**		
	Grade 5 (11-12 years)	23 (29)
	Grade 8 (14-16 years)	33 (44)
	Grade 11 (17-18 years)	22 (28)
**Parental education, n (%)**		
	Higher level^a^	39 (50)
	Lower level	30 (38)
	Missing information	9 (12)
Overweight, obese^b^, n (%)		15 (19)
**Plasma carotenoid concentration (µmol/L, n=73), median (p25, p75)^c^**		
	Alpha-carotene,	0.19 (0.13, 0.38)
	Beta-carotene	0.79 (0.52, 1.04)
	Beta-cryptoxantin	0.14 (0.11, 0.19)
	lutein+zeaxantin	0.36 (0.26, 0.47)
	Lycopene	0.86 (0.71, 1.06)
Total carotenoids, median (p25, p75)		2.39 (1.97, 3.03)
**Plasma** **alkylresorcinol concentration** **(nmol/L, n=72), median (p25, p75)**		
	C:17	3.2 (1.3, 6.1)
	C:19	15.8 (9.0, 32.3)
	C:21	24.8 (12.9, 48.5)
	C:23	6.1 (2.4, 11.7)
	C:25	3.7 (1.5, 8.2)
	Total alkylresorcinols	51.1 (29.9, 101.6)
	C17:0/C21:0	0.16 (0.08, 0.25)
**Physical activity (counts/min,** **n=60), median (p25, p75)**		376 (289, 492)

^a^At least one parent had postsecondary education.

^b^Based on Cole and Lobstein [39].

^c^25th and 75th percentiles.

### Reported Intake by RiksmatenFlexDiet and Recall Interviews

Reported intakes of energy, macronutrients, beta-carotene, whole grain wheat and rye, vegetable and fruit by RiksmatenFlexDiet and recall interviews are presented in Table 2. Reported energy and macronutrient intake was higher with RiksmatenFlexDiet than with recall interviews. However, intake of beta-carotene, whole grain wheat and rye, vegetables, and fruits did not differ between the methods.

**Table 2:** Reported dietary intake by RiksmatenFlex and recall interviews (n=78).

Dietary intake/day^a^	RiksmatenFlexDiet, mean (SD)	Recall interviews, mean (SD)	*P* value^b^	Intraclass correlation (95% CI)
Energy, MJ	8.92 (2.77)	8.04 (2.67)	.01	0.53 (0.35-0.67)
Protein, g	85 (33)	74 (24)	<.001	0.57 (0.39-0.70)
Fat, g	86 (30)	76 (33)	.01	0.27 (0.05-0.47)
Carbohydrates, g	243 (85)	225 (78)	.04	0.57 (0.40-0.70)
Dietary fiber, g	20 (10)	17 (6.0)	.01	0.45 (0.26-0.61)
Whole grain wheat and rye, g	12.4 (13.2)	12.0 (13.1)	.82	0.29 (0.07-0.48)
Beta-carotene, mg	1975 (2415)	1876 (2359)	.71	0.50 (0.32-0.65)
Vegetables, g	137 (106)	139 (95)	.90	0.23 (0.01-0.43)
Fruits, g	87 (112)	88 (98)	.89	0.56 (0.39-0.70)
Fruits and vegetables, g	224 (169)	227 (150)	.88	0.49 (0.30-0.64)

^a^Mean of two days

^b^Paired *t* test.

To investigate the agreement between the two methods, Bland-Altman plots were used for energy and macronutrient intake (Multimedia Appendix 3). For all comparisons, a few outliers were noted, but the majority fell within the limits of agreement. The intraclass correlations between the two methods were moderate and ranged from 0.57 for protein to 0.23 for vegetables. The strength of agreement for reported energy intake and macronutrient intake was fair to moderate. For energy, 87% were classified into the same or the adjacent tertile, and the weighted kappa was 0.42 (95% CI 0.25-0.58). Corresponding figures for protein, fat, and carbohydrates were 91% and 0.36 (95% CI 0.19-0.53), 86% and 0.22 (95% CI 0.04-0.39), 96% and 0.51 (95% CI 0.36-0.66), respectively.

### Reported Energy Intake and Estimated Energy Expenditure

**Figure 6:**
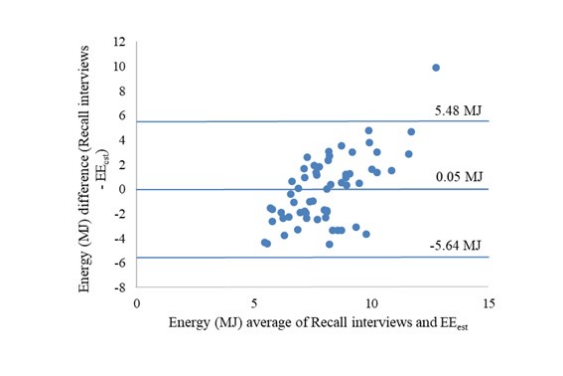
Bland-Altman plot of energy intake by recall interviews and estimated energy expenditure (n=60). EEest: estimated total energy expenditure.

Mean accelerometer-estimated energy expenditure was 8.13 MJ (95% CI 7.80-8.46) compared with the mean reported energy intake of 8.80 (95% CI 8.19-9.42) and 8.05 (7.36-8.74) by RiksmatenFlexDiet and Recall interviews, respectively. Estimated energy expenditure was overestimated by RiksmatenFlexDiet (*P*=.02) but not by the recall interviews (*P*=.82). The agreement between reported energy intake and estimated energy expenditure for the two methods was also examined in Bland-Altman plots (Figures 5 and 6). The limits of agreement were wider for the comparison with recall interviews than for RiksmatenFlexDiet. Both plots show a bias toward overestimation at higher means. The Spearman correlation between estimated energy expenditure and reported energy intake was 0.34 (*P*=.008) for RiksmatenFlexDiet and 0.16 (*P*=.21) for recall interviews.

### Reported Intake Versus Biomarkers

The Spearman correlations between the alkylresorcinol concentrations and reported intake of whole grain wheat and rye by both dietary methods were moderate but statistically significant for all comparisons (Table 3). The correlations were consistently higher for RiksmatenFlexDiet than for recall interviews.

The correlations between carotenoids and intake of vegetables and fruit were weak for both methods (Table 4). The strongest correlation was observed for lutein/zeaxanthin and intake of fruit and vegetables for RiksmatenFlexDiet (r=0.46; *P*<.001).

**Table 3:** Spearman rank correlations between energy-adjusted intake (g/MJ) of whole grain wheat and rye and plasma alkylresorcinol concentrations (n=72).

Alkylresorcinol	RiksmatenFlexDiet		Recall interviews	
	ρ^a^	*P* value	ρ	*P* value
C:17	0.43	<.001	0.32	.006
C:19	0.37	.001	0.29	.01
C:21	0.31	.008	0.26	.26
C:23	0.34	.004	0.27	.02
C:25	0.37	.001	0.29	.01
Total alkylresorcinols	0.36	.002	0.29	.01

^a^Spearman rank correlation coefficient.

**Table 4:** Spearman rank correlations between energy-adjusted intake (g/MJ) of fruit and vegetables and beta-carotene and plasma carotenoid concentrations (n=73).

Carotenoid		Vegetables		Fruits		Fruits and vegetables		Beta-carotene	
		ρ^a^	*P* value	ρ	*P* value	ρ	*P* value	ρ	*P* value
**RiksmatenFlexDiet**									
	Alpha-carotene	–0.04	.72	0.22	.06	0.09	.47	0.28	.01
	Beta-carotene	–0.01	.95	0.15	.20	0.07	.58	0.22	.06
	Beta-cryptoxanthin	0.00	.98	0.17	.14	0.11	.36	0.12	.30
	Lutein/zeaxanthin	0.37	.001	0.28	.02	0.46	<.001	0.16	.19
	Lycopene	0.28	.02	0.05	.68	0.21	.08	0.25	.03
	Total carotenoids	0.15	.21	0.15	.21	0.19	.11	0.32	.006
**Recall interviews**									
	Alpha-carotene	–0.19	.11	0.21	.08	–0.01	.95	0.28	.02
	Beta-carotene	–0.06	.59	0.08	.49	0.02	.90	0.26	.03
	Beta-cryptoxanthin	–0.10	.39	0.06	.60	–0.06	.61	–0.03	.80
	Lutein/zeaxanthin	0.29	.01	0.24	.04	0.28	.02	0.03	.81
	Lycopene	0.17	.15	0.01	.91	0.11	.36	0.07	.56
	Total carotenoids	–0.00	.97	0.12	.33	0.06	.62	0.21	.07

^a^Spearman rank correlation coefficient.

## Discussion

### Principal Findings and Comparison With Previous Work

The results of this study suggest that RiksmatenFlexDiet provides dietary intake estimates comparable to recall interviews in adolescents. RiksmatenFlexDiet, but not recall interviews, overreported energy intake compared with the estimated energy expenditure, but the limits of agreement were wider for the comparison with recall interviews than for RiksmatenFlexDiet. The ability to rank intake by estimated energy expenditure and biomarker concentrations was modest for both methods, but the correlations between intake and the reference methods were generally stronger for RiksmatenFlexDiet than for the recall interviews.

Reported energy intake was higher by RiksmatenFlexDiet than by recall interviews, and consequently, intake of macronutrients was also higher. In studies of adolescents, where energy intake by computer or Web-based 24-hour dietary recall methods have been compared with 24-hour dietary recall interviews, the difference between the methods have generally been small [15,44,45]. Limits of agreement for energy intake and macronutrients were wide in our study but similar to those observed in earlier studies of adolescents [44] or wider [15]. Agreement and ability to rank dietary intake were also better with these methods [15,44] compared with RiksmatenFlexDiet. However, in contrast to our study where dietary intake by the two methods was assessed on different days, the reference method in these studies covered the same days, and a lower agreement and ranking ability would therefore be expected when different days are compared. When a Web-based 24-hour dietary recall was compared with food diaries, thus covering different time periods, limits of agreement, agreement, and ability to rank were comparable to the results of our study [46].

The agreement and ability to rank individuals by energy intake was better for RiksmatenFlexDiet than for the recall interviews. The Bland-Altman plots showed large variation at the individual level for both methods, as generally seen in validation studies of energy intake [47], and this was also expected in our study, as 2 days is not enough to estimate habitual energy intake. The limits of agreement, which were narrower for RiksmatenFlexDiet than for recall interviews, were in line with an earlier study of a Web-based food recall where energy intake was underestimated [21], but wider than that reported for a Web-based food diary in 10-year-old children [20]. The modest correlation between energy intake and estimated energy expenditure for RiksmatenFlexDiet was similar to other Web-based tools [20,21,26]. Although a somewhat stronger correlation between energy intake by two 24-hour dietary recall interviews has been reported earlier [26], this correlation was not significant for recall interviews in our study.

The evaluation of energy intake with estimated energy expenditure from accelerometer data should be interpreted with caution, since the energy expenditure estimation is not without errors. Furthermore, the comparison between energy intake and energy expenditure assumes energy balance, which may not be the case in growing adolescents. In addition, the equations used for estimating total energy expenditure were developed in a small group of 9-year-old children [40] and may be less accurate in adolescents [21]. Finally, activities less well captured by accelerometers, for example, biking, may be more common in adolescents, and some participants may also have taken off their accelerometers during intensive ball sports. The registered counts per minute were quite low in this study compared with European adolescents [48] and a subgroup from the Riksmaten adolescents 2016-2017 survey [49]. Thus, it is possible that energy expenditure is underestimated and that RiksmatenFlexDiet, as suggested by the comparison, does not overreport energy intake on an average.

The correlations between whole grain wheat and rye intake and alkylresorcinol homologs were acceptable for both methods and in line with other studies on children and adults [23,34,50,51]. Intake of bread and breakfast cereals, the major sources of whole grain wheat and rye in Swedish adolescents [36], is difficult to report. Photos of these foods are linked to the search engine in RiksmatenFlexDiet, to help the participants find the food closest to what they have eaten. This may have resulted in stronger correlations for RiksmatenFlexDiet than for recall interviews.

Reported intake of fruit and vegetables was similar by the two methods. The intraclass correlation between the two methods was stronger for fruit than for vegetables, in line with an earlier study on adolescents [15]. The correlations between intake of fruit and vegetables and the objective biomarkers plasma carotenoids were, however, weak for both methods. Moderate correlations between several carotenoids and various dietary assessment methods have been reported in adults [34,52-55], adolescent girls (but not boys) [56], and children and adolescents [22]. However, stronger correlations between intake from a Web-based food diary and carotenoids have also been reported in 10-year-old children [19]. Significantly stronger correlations in younger children (8-9 years old) compared to older children (12-14 years old) have also been reported [22]. The strongest correlations in our study were seen for lutein-zeaxanthin by both methods, with the strongest correlation for RiksmatenFlexDiet. This is in line with the correlations presented for adult women, but not men, where all carotenoid concentrations were significantly correlated with fruit and vegetable intake by a Web-based 4-day food diary [34]. Food records may also produce stronger correlations between intake and plasma concentrations than recall methods [32,46].

### Strengths and Limitations

A major strength of this study is that dietary intake has been validated against objectively measured physical activity and biomarkers. Estimating energy expenditure from equations and accelerometer data is not without errors, as previously discussed, but the physical activity measurements are objectively measured. Biomarker concentrations provide a reflection of dietary intake, but perfect agreement could not be expected because genetic variability, lifestyle, and physiological factors influence the concentrations in plasma [27]. Furthermore, neither 2 days of dietary intake nor a single blood sample may reflect habitual intake of whole grain wheat and rye, fruits, and vegetables. Another possible limitation is that blood samples were not drawn in the fasting state. However, fasting was not a confounding factor in validation studies of carotenoid concentrations and dietary carotenoid intake [32], and correlations between alkylresorcinol concentrations and wholegrain wheat and rye intake were similar to those reported in other studies [23,34,50,51].

Another strength is that the comparison and validation of RiksmatenFlexDiet were performed in adolescents by using a recruitment procedure similar to the national dietary survey of adolescents that RiksmatenFlexDiet was intended for. Although this study was not designed to obtain a representative sample, the geographical spread across Sweden was good. Furthermore, around 50% of the participants came from homes where at least one parent had a postsecondary education. This proportion is lower than that in the subsequent national dietary survey in adolescents (60%), but higher than expected in households with children in the general population (38%) [36]. Thus, we believe that the results are generalizable to the population that the method is intended for. Moreover, we are not dependent on a convenience sample of self-selected and motivated participants that many comparison and validation studies rely on. However, the study is limited by the large proportion of participants who did not complete the random second registration day, making subanalyses by gender and age difficult. This day was lost for many participants, as they, particularly those in the youngest age group, did not read their email prompts and consequently missed the requirement of recording on their last day. In the main national dietary survey, the date of the randomly selected day was therefore visible when the participants logged on to RiksmatenFlex the first time. In addition, text message prompts and reminders were introduced. Thus, participants knew about the last day in advance, and the reminders also reached them. Knowing which day to record in advance could potentially influence how the participants report their diet, but this limitation was considered less problematic for the final dietary information as compared to participants not completing their last day. With these changes, almost 90% of the participants completed the last random day in the main survey [36].

In general, Web-based methods are well accepted by participants [15,46]. How well computer-based methods work may depend on the structure of the method. Many foods and details may make a method difficult and laborious to complete. Some computer and Web-based methods include a large number of different foods [7,15], while others focus on fewer generic core foods [46]. A shorter list of generic foods, as in RiksmatenFlexDiet, makes the method more user-friendly, but as reported previously [46], some level of detail was inevitably lost. A future challenge is to develop Web-based methods that capture as much detail as possible without complicating completion too much.

### Conclusions

This study demonstrates that RiksmatenFlex provides dietary intake estimates that are comparable with estimates from 24-hour dietary recall interviews in Swedish adolescents. The tool can easily be adapted to other age groups by changing food list and portion sizes. Thus, RiksmatenFlex has great potential for cost-effective dietary data collections in upcoming national dietary surveys and other studies in Sweden. Thus far, computer and Web-based tools do not provide dietary information with less bias than traditional dietary assessment methods; however, new technology has the potential to provide solutions that could further reduce dietary reporting bias. This is an important area for future research.

## Appendix

Multimedia Appendix 1 Photos of bread, ready-to-eat sandwiches, cereals, ice cream, and fat spreads attached to the search engine and food list.

Multimedia Appendix 2 The food list in RiksmatenFlexDiet.

Multimedia Appendix 3 Bland-Altman plots of reported intake of energy and macronutrients between the two dietary assessment methods.

## Abbreviations

Cpm: counts per minute
EEest: estimated total energy expenditure
EFSA: European Food Safety Authority
NFA: National Food Agency, Sweden
OEMC: Occupational and Environmental Medicine Centers
