# Supplementary material for: Use of a Web-Based Dietary Assessment Tool (RiksmatenFlex) in Swedish Adolescents: Comparison and Validation Study
Source: J Med Internet Res. 2019 Oct 4;21(10):e12572. doi: 10.2196/12572 (PMC6914230; doi:10.2196/12572)
Supplement: Multimedia Appendix 1 [file jmir_v21i10e12572_app1.pdf]

Multimedia Appendix 1. Photos attached to the food list in RiksmatenFlexDiet.

Bread

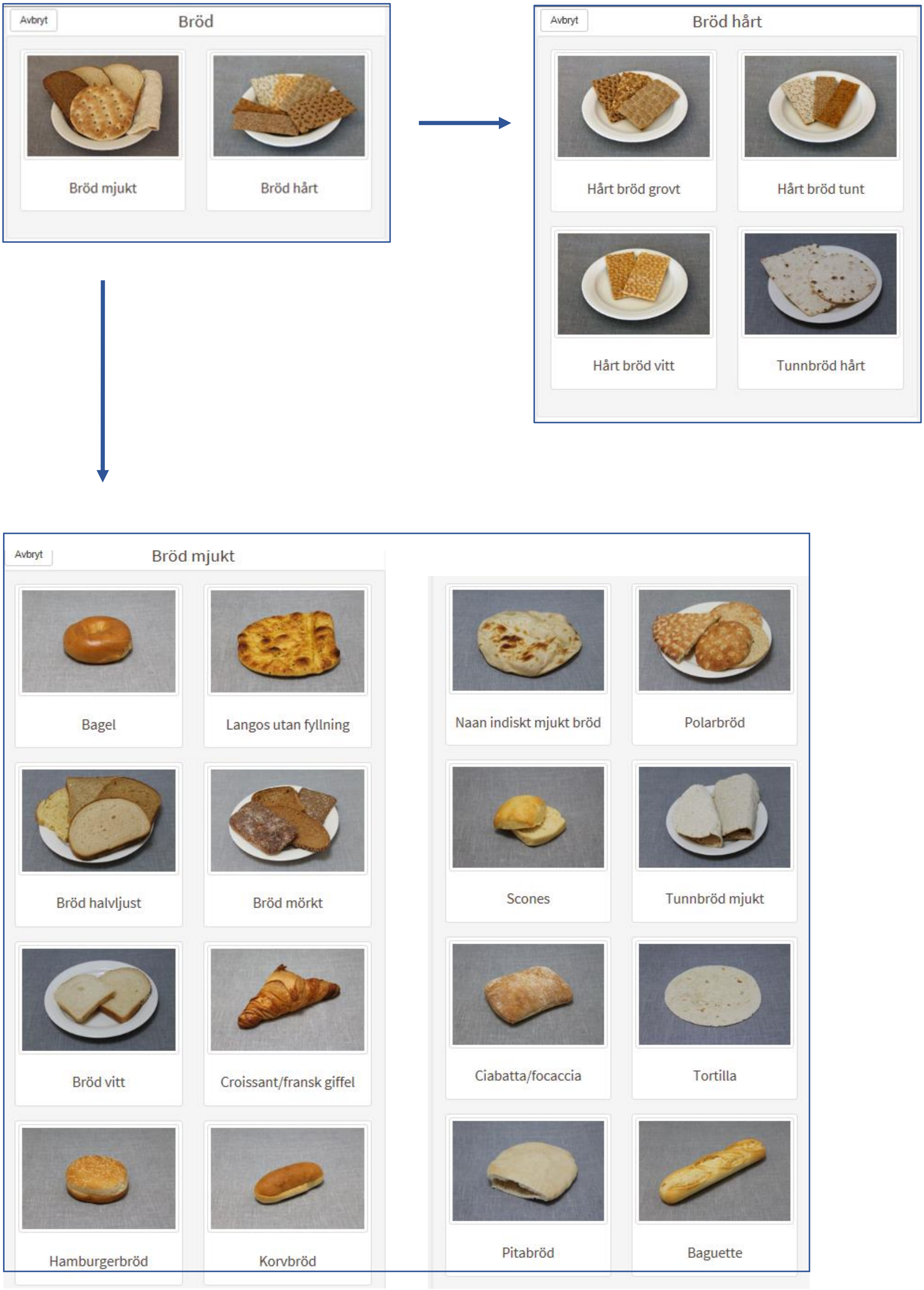

Ready to eat sandwiches

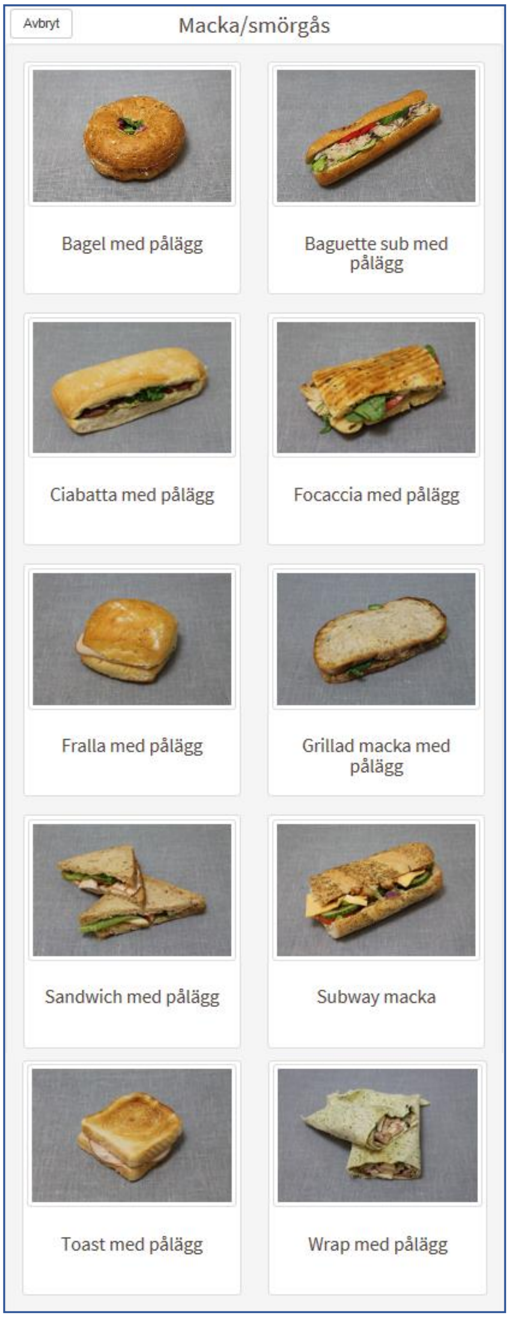

Cereals

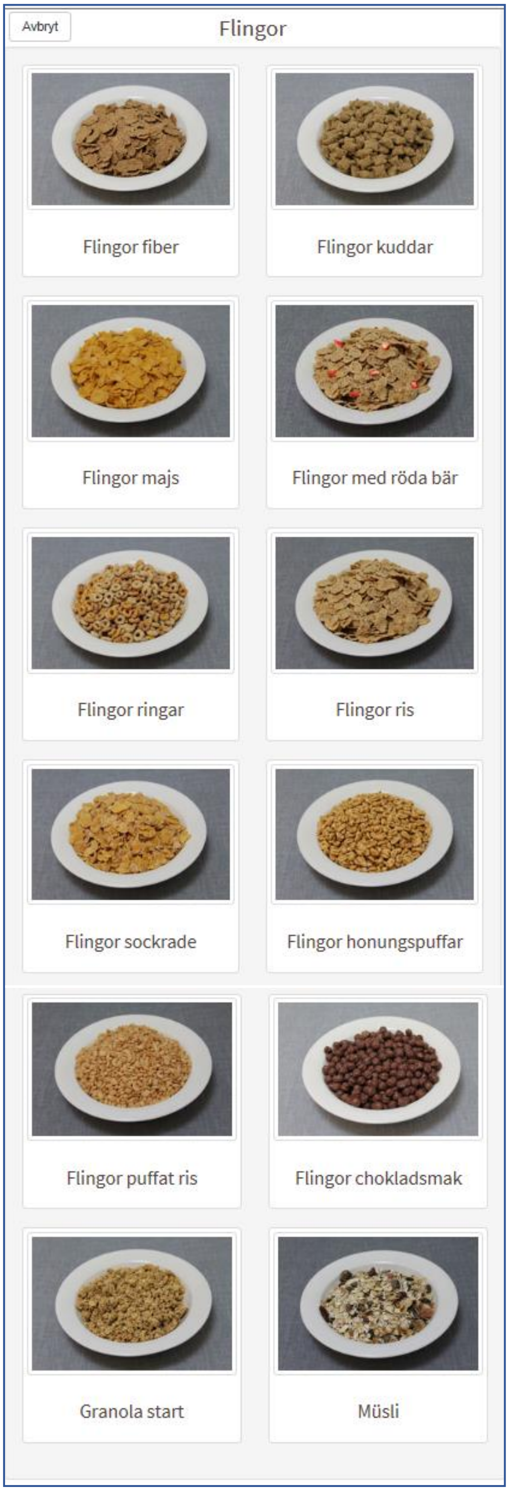

Ice cream

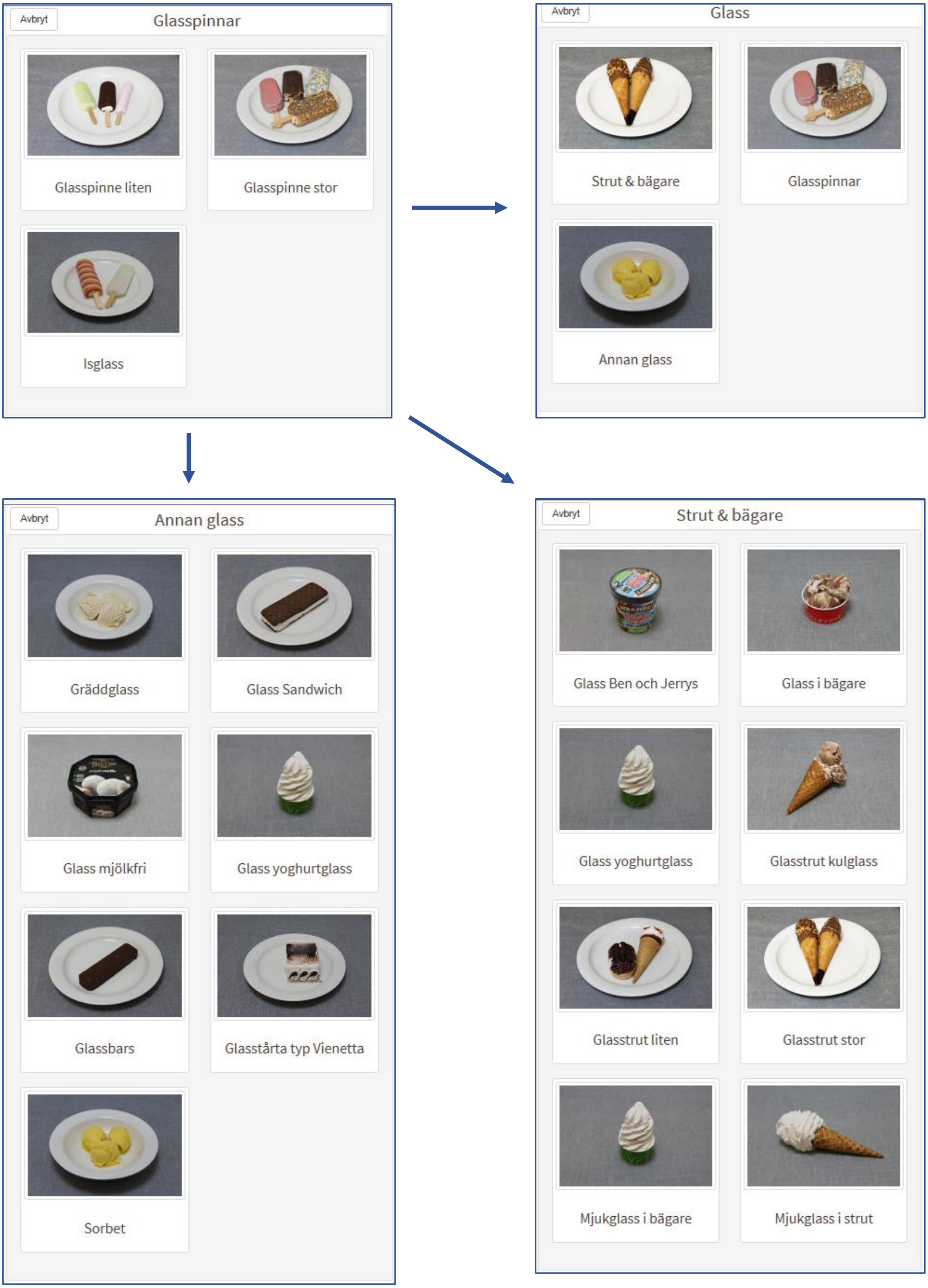
Avbryt
Glasspinnar
Glasspinne liten
Glasspinne stor
Isglass
Avbryt
Glass
Strut & bägare
Glasspinnar
Annan glass
Avbryt
Annan glass
Gräddglass
Glass Sandwich
Glass mjölkfri
Glass yoghurtglass
Glassbars
Glasstårta typ Vienetta
Sorbet
Avbryt
Strut & bägare
Glass Ben och Jerrys
Glass i bägare
Glass yoghurtglass
Glasstrut kulglass
Glasstrut liten
Glasstrut stor
Mjukglass i bägare
Mjukglass i strut

Fat spreads

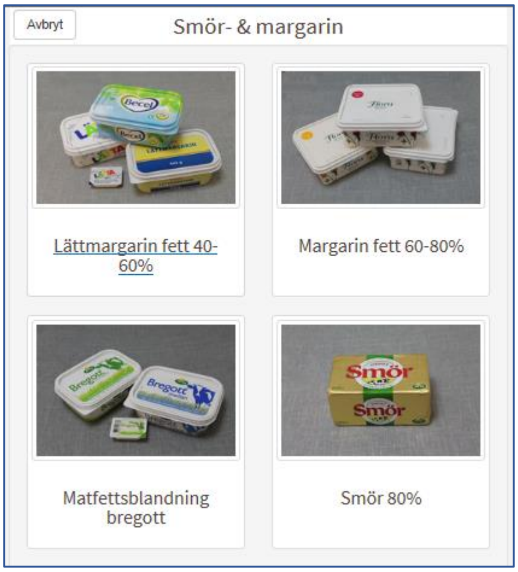
Avbryt
Smör- & margarin
Lättmargarin fett 40-60%
Margarin fett 60-80%
Matfettsblandning bregott
Smör 80%
