# Supplementary material for: Use of a Web-Based Dietary Assessment Tool (RiksmatenFlex) in Swedish Adolescents: Comparison and Validation Study
Source: J Med Internet Res. 2019 Oct 4;21(10):e12572. doi: 10.2196/12572 (PMC6914230; doi:10.2196/12572)
Supplement: Multimedia Appendix 2 [file jmir_v21i10e12572_app2.pdf]

Number	Food item (Swedish)	Food item	Main food group (Swedish)	Main food group	Study[a]
536	Kelp	Kelp dried	Algprodukter	Algea	1
3973	Bönor kokta konserv	Beans tinned	Baljväxter (bönor, linser och ärter)	Leguminous plant	1
5742	Groddar	Sprouts unspec.	Baljväxter (bönor, linser och ärter)	Leguminous plant	1
5741	Gröna bönor ospec	Green beans unspec.	Baljväxter (bönor, linser och ärter)	Leguminous plant	1
373	Gröna ärtor	Green peas	Baljväxter (bönor, linser och ärter)	Leguminous plant	1
3815	Kikärter konserv	Chickpeas canned	Baljväxter (bönor, linser och ärter)	Leguminous plant	1
3926	Linser kokta med salt	Lentils dried boiled w/ salt	Baljväxter (bönor, linser och ärter)	Leguminous plant	1
3308	Sockerärtor kokta m salt	Sugar pea boiled w/ salt	Baljväxter (bönor, linser och ärter)	Leguminous plant	1
1431	Blodpudding blodkorv fett 13% stekt	Black pudding fat 13% fried	Blodprodukter blodrätter	Blood products and dishes	1
1433	Paltbröd kokt	Blood bread boiled	Blodprodukter blodrätter	Blood products and dishes	1
1224	Hönsbuljong ätfärdig	Chicken stock RTE	Buljong	Bouillon	1
1621	Amerikanska cookies choklad	American chocolate cookies	Bullar kakor tårtor mm	Buns cakes cookies	1
1677	Amerikanska muffins ospec	American muffins unspec.	Bullar kakor tårtor mm	Buns cakes cookies	1
5833	Brownie	Brownie	Bullar kakor tårtor mm	Buns cakes cookies	1
1888	Chokladbollar	Chocolate balls wholemeal	Bullar kakor tårtor mm	Buns cakes cookies	1
5612	Daimtårta	Daim cake	Bullar kakor tårtor mm	Buns cakes cookies	1
5582	Dubbla kex kaka med fyllning	Double biscuit	Bullar kakor tårtor mm	Buns cakes cookies	1
5580	Enkla kex kakor små	Biscuit small	Bullar kakor tårtor mm	Buns cakes cookies	1
5581	Enkla kex kakor stora	Biscuit large	Bullar kakor tårtor mm	Buns cakes cookies	1
1602	Fyllt vetebröd bullar längd ospec.	Sweet wheat bread filled	Bullar kakor tårtor mm	Buns cakes cookies	1
5611	Gräddtårta ospec	Cream cake	Bullar kakor tårtor mm	Buns cakes cookies	1
5607	Kanelgiffel	Cinnamon bun crescent	Bullar kakor tårtor mm	Buns cakes cookies	1
5583	Kex kaka med chokladöverdrag	Biscuit w/ chocolate coating	Bullar kakor tårtor mm	Buns cakes cookies	1
3591	Kladdkaka	Chocolate mud cake	Bullar kakor tårtor mm	Buns cakes cookies	1
5610	Konditorbit	Mazarin bisque punch roll	Bullar kakor tårtor mm	Buns cakes cookies	1
1638	Mandelbiskvier	Almond macaroons	Bullar kakor tårtor mm	Buns cakes cookies	1
1626	Maräng	Meringue	Bullar kakor tårtor mm	Buns cakes cookies	1
4200	Marängtårta	Meringue cake (Pavlova)	Bullar kakor tårtor mm	Buns cakes cookies	1
1678	Mjuk kaka sockerkaka mjuk pepparkaka	Sponge cake gingerbread cake	Bullar kakor tårtor mm	Buns cakes cookies	1
2129	Morotskaka	Carrot cake	Bullar kakor tårtor mm	Buns cakes cookies	1
3055	Muffins	Muffin queen cake	Bullar kakor tårtor mm	Buns cakes cookies	1
1880	Mums-mums	Mums-mums raw meringue on wafer covered w/ chocolate	Bullar kakor tårtor mm	Buns cakes cookies	1
5608	Munk ospec	Donut unspec.	Bullar kakor tårtor mm	Buns cakes cookies	1
1624	Pepparkakor	Gingerbread biscuits	Bullar kakor tårtor mm	Buns cakes cookies	1
1648	Prinsesstårta fylld m grädde vaniljkräm hallonsylt marsipan	Layer cake filled w/ whipped cream vanilla custard raspberry jam covered w/ marzipan	Bullar kakor tårtor mm	Buns cakes cookies	1
1610	Saffransbröd	Sweet wheat bread w/ saffron	Bullar kakor tårtor mm	Buns cakes cookies	1
1668	Semla	Sweet wheat bun filled w/ whipped cream and almond paste	Bullar kakor tårtor mm	Buns cakes cookies	1

[a] Foods in validation study and main survey Riksmaten ungdom 2016-17 = 1; Foods in main survey, only = 2; Foods in validation study, only = 3

Number	Food item (Swedish)	Food item	Main food group (Swedish)	Main food group	Study[a]
1594	Sött vetebröd kanelbullar hembakta	Sweet wheat bread cinnamon buns home-made	Bullar kakor tårtor mm	Buns cakes cookies	1
1612	Wafers fyllda rån kex	Filled wafers biscuits	Bullar kakor tårtor mm	Buns cakes cookies	1
1603	Veteskorpor	Wheat rusks	Bullar kakor tårtor mm	Buns cakes cookies	1
1608	Wienerbröd fyllt m vaniljkräm sylt florsocker	Danish pastry filled w/ vanilla custard jam powdered sugar	Bullar kakor tårtor mm	Buns cakes cookies	1
554	Björnbär	Blackberries	Bär färska frysta	Berries fresh frozen	1
555	Blåbär	Blueberries	Bär färska frysta	Berries fresh frozen	1
594	Bär färska	Berries fresh	Bär färska frysta	Berries fresh frozen	1
523	Hallon	Raspberries	Bär färska frysta	Berries fresh frozen	1
526	Jordgubbar	Strawberries	Bär färska frysta	Berries fresh frozen	1
2843	Vinbär	Currant	Bär färska frysta	Berries fresh frozen	1
1587	Chips smaksatta fett 34%	Crisps flavoured 34% fat	Chips popcorn o dyl	Crisps, popcorn, etc.	1
3238	Frasiga figurer typ skruvar (snacks)	Crispy potato snacks	Chips popcorn o dyl	Crisps, popcorn, etc.	1
1589	Japanmix nötter typ Exotic snacks	Mixed savory snacks	Chips popcorn o dyl	Crisps, popcorn, etc.	1
1580	Jordnötsbågar jordnötsringar	Peanut doodles or rings	Chips popcorn o dyl	Crisps, popcorn, etc.	1
1582	Ostbågar	Cheese doodles (cheese-flavored cheese puffs)	Chips popcorn o dyl	Crisps, popcorn, etc.	1
1586	Popcorn mikro fett 22%	Popcorn microwave fat 22%	Chips popcorn o dyl	Crisps, popcorn, etc.	1
4072	Räkchips	Crisps shrimp taste	Chips popcorn o dyl	Crisps, popcorn, etc.	1
1585	Salta pinnar	Salted sticks	Chips popcorn o dyl	Crisps, popcorn, etc.	1
1581	Tortilla chips	Corn crisps	Chips popcorn o dyl	Crisps, popcorn, etc.	1
5600	Choklad lösgodis	Chocolate bulk confectionary	Choklad	Chocolate	1
5605	Choklad med fyllning ospec	Chocolate w/ toffee center unspec.	Choklad	Chocolate	1
5702	Mjölkchoklad	Milk chocolate unspec.	Choklad	Chocolate	1
2052	Mörk choklad kakao >70 %	Dark chocolate cocoa >70%	Choklad	Chocolate	1
5152	Alkoläsk kolsyrad dryck vol. % 4-5	Soft drink w/ alcohol vol. % 4-5	Cider alkoläsk drink	Cider, soft drink with alcohol, drinks	1
3288	Cider 2,25 %	Cider vol. % 2.25	Cider alkoläsk drink	Cider, soft drink with alcohol, drinks	1
3111	Cider med ca 4.5 % alkohol	Cider vol. % 4.5	Cider alkoläsk drink	Cider, soft drink with alcohol, drinks	1
1929	Irish coffee	Irish Coffee	Cider alkoläsk drink	Cider, soft drink with alcohol, drinks	1
4256	Long drink - starksprit (ca 4-6 cl) blandat med läsk el juice	Long drink (ca 4-6 cl spirits)	Cider alkoläsk drink	Cider, soft drink with alcohol, drinks	1
4260	Vinbål	Punch w/ wine	Cider alkoläsk drink	Cider, soft drink with alcohol, drinks	1
105	Camembert vitmögelost lätt fett 11%	Camembert cheese white mould ripened low fat fat 11%	Dessertost	Soft cheese	1

[a] Foods in validation study and main survey Riksmaten ungdom 2016-17 = 1; Foods in main survey, only = 2; Foods in validation study, only = 3

Number	Food item (Swedish)	Food item	Main food group (Swedish)	Main food group	Study[a]
111	Getost chèvre vitmögelost fett 25%	Cheese chèvre french goat milk white mold ripened fat 25%	Dessertost	Soft cheese	1
2900	Ost dessertost	Cheese for dessert	Dessertost	Soft cheese	1
3267	Baklava	Baklava	Efterrätter	Dessert	1
1784	Cheesecake m botten av digestivekex	Cheese cake w/ crust made of digestive biscuits	Efterrätter	Dessert	1
1764	Chokladpudding	Chocolate fool or chocolate pudding	Efterrätter	Dessert	1
1775	Citronfromage	Lemon mousse	Efterrätter	Dessert	1
732	Fattiga riddare	Bread slices dipped in egg batter fried ('poor knights')	Efterrätter	Dessert	1
1790	Friterad banan	Deep fried banana	Efterrätter	Dessert	1
5848	Frukt bär m vit choklad gratinerat	Fruit berries w/ white chocolate gratinated	Efterrätter	Dessert	1
1761	Marängsviss hovdessert	Meringues and whipped cream decorated w/ chocolate sauce 'court dessert'	Efterrätter	Dessert	1
2184	Ostkaka fett 7%	Swedish cheese cake fat 7%	Efterrätter	Dessert	1
1787	Paj efterrätt ospec	Sweet pie (pie as a dessert unspec.)	Efterrätter	Dessert	1
3008	Pannacotta	Vanilla pudding Panna cotta	Efterrätter	Dessert	1
1781	Rabarberpaj	Rhubarb pie	Efterrätter	Dessert	1
838	Ris à la Malta	Glorified rice	Efterrätter	Dessert	1
839	Risgrynskaka el risgrynspudding	Rice pudding	Efterrätter	Dessert	1
2886	Tiramisu	Tiramisu	Efterrätter	Dessert	1
1791	Ugnsstekt äpple	Baked apples	Efterrätter	Dessert	1
1782	Äppelpaj	Apple pie	Efterrätter	Dessert	1
5691	Portionsyoghurt	Small yoghurt pot size	Fil yoghurt o syrade produkter	Fermented milk products	1
5698	Portionsyoghurt med müsli	Small yoghurt pot size w/ müsli	Fil yoghurt o syrade produkter	Fermented milk products	1
1271	Sardiner i olja konserv	Sardines canned in oil	Fisk färsk fryst kokt	Fish fresh frozen cooked	1
2941	Vit fisk kokt	White fish boiled	Fisk färsk fryst kokt	Fish fresh frozen cooked	1
2944	Övrig fisk kokt	Other fish boiled	Fisk färsk fryst kokt	Fish fresh frozen cooked	1
5751	Skaldjur ospec	Shellfish unspec.	Fisk o skaldjur	Fish and shellfish	1
5746	Tonfisk konserv	Tuna canned	Fisk o skaldjur	Fish and shellfish	1
1306	Fisk friterad	Fish fritters or deep fried fish	Fisk o skaldjursprodukter o rätter	Fish and shellfish dishes	1
1371	Fisk m mandel o ströbröd fryst	Fish w/ almonds bread crumbs frozen	Fisk o skaldjursprodukter o rätter	Fish and shellfish dishes	1
2948	Fisk och skaldjursgryta	Fish and shellfish casserole	Fisk o skaldjursprodukter o rätter	Fish and shellfish dishes	1
1345	Fiskbullar m hummersås konserv tillagad	Fish balls w/ lobster sauce canned prepared	Fisk o skaldjursprodukter o rätter	Fish and shellfish dishes	1
1351	Fiskbullar panerade stekta	Fish balls canned breaded fried	Fisk o skaldjursprodukter o rätter	Fish and shellfish dishes	1
5502	Fiskgratäng	Fish gratin	Fisk o skaldjursprodukter o rätter	Fish and shellfish dishes	1
1358	Fiskgratäng m dillsås	Fish au gratin w/ dill sauce mashed potatoes	Fisk o skaldjursprodukter o rätter	Fish and shellfish dishes	1
1372	Fiskgratäng m potatismos o dillsås fryst tillagad	Fish au gratin w/ mashed potatoes dill sauce frozen prepared	Fisk o skaldjursprodukter o rätter	Fish and shellfish dishes	1
1415	Fiskpastej	Fish paste/paté	Fisk o skaldjursprodukter o rätter	Fish and shellfish dishes	1

[a] Foods in validation study and main survey Riksmaten ungdom 2016-17 = 1; Foods in main survey, only = 2; Foods in validation study, only = 3

Number	Food item (Swedish)	Food item	Main food group (Swedish)	Main food group	Study[a]
1356	Fiskpaté m räkor	Fish paté w/ shellfish	Fisk o skaldjursprodukter o rätter	Fish and shellfish dishes	1
1294	Fiskpinnar stekta	Fish fingers breaded, fried	Fisk o skaldjursprodukter o rätter	Fish and shellfish dishes	1
1406	Friterade räkor restaurang	Shrimps deep fried restaurant	Fisk o skaldjursprodukter o rätter	Fish and shellfish dishes	1
3037	Gubbröra	Mix of egg, anchovy, red onion, creme fraiche, mayonnaise (indiviudal recipes may differ)	Fisk o skaldjursprodukter o rätter	Fish and shellfish dishes	1
3500	Inlagd sill	Pickled herring	Fisk o skaldjursprodukter o rätter	Fish and shellfish dishes	1
3035	Inlagd sill m gräddfilssås	Pickled herring w/ sour cream	Fisk o skaldjursprodukter o rätter	Fish and shellfish dishes	1
3034	Inlagd sill med tomatsås	Pickled herring w/ tomato sauce	Fisk o skaldjursprodukter o rätter	Fish and shellfish dishes	1
5880	Krustader fyllda skagenröra svampstuvning	Croustades filled w/ mushroom stew or skagen mixture	Fisk o skaldjursprodukter o rätter	Fish and shellfish dishes	1
258	Laxpudding	Cured salmon and potatoes baked in eggs	Fisk o skaldjursprodukter o rätter	Fish and shellfish dishes	1
1296	Makrillfilé konserv i tomatsås	Mackerel fillets canned in tomato sauce	Fisk o skaldjursprodukter o rätter	Fish and shellfish dishes	1
3966	Plattfisk panerad stekt	Flatfish breaded pan-fried	Fisk o skaldjursprodukter o rätter	Fish and shellfish dishes	1
1411	Räkstuvning	Shrimps in thick white sauce	Fisk o skaldjursprodukter o rätter	Fish and shellfish dishes	1
1299	Salt sill panerad stekt hemlagad	Herring salted coated fried home-made	Fisk o skaldjursprodukter o rätter	Fish and shellfish dishes	1
1272	Sardiner i tomatsås konserv	Sardines canned in tomato sauce	Fisk o skaldjursprodukter o rätter	Fish and shellfish dishes	1
4610	Senapssill u sås	Pickled herring mustard sauce drained	Fisk o skaldjursprodukter o rätter	Fish and shellfish dishes	1
1328	Sill panerad stekt	Herring breaded fried	Fisk o skaldjursprodukter o rätter	Fish and shellfish dishes	1
259	Sillpudding	Salted herring and potatoes baked in eggs	Fisk o skaldjursprodukter o rätter	Fish and shellfish dishes	1
1297	Surströmming	Baltic herring fermented	Fisk o skaldjursprodukter o rätter	Fish and shellfish dishes	1
1368	Sushi olika sorter	Sushi diff. kinds	Fisk o skaldjursprodukter o rätter	Fish and shellfish dishes	1
3896	Tonfisk och tomatsås	Tuna and tomato sauce	Fisk o skaldjursprodukter o rätter	Fish and shellfish dishes	1
2943	Vit fisk panerad och stekt	White fish breaded and pan-fried	Fisk o skaldjursprodukter o rätter	Fish and shellfish dishes	1
5789	Lax kallrökt el gravad	Salmon cold smoked or dry salted	Fisk rökt	Fish smoked	1
1288	Lax urtagen varmrökt	Salmon drawn smoked	Fisk rökt	Fish smoked	1
1316	Lax stekt	Salmon fried	Fisk stekt ej panerad	Fish fried	1
1333	Strömming filé stekt	Baltic herring fried	Fisk stekt ej panerad	Fish fried	1
1303	Ål ugnsstekt hemlagad	Eel baked home-made	Fisk stekt ej panerad	Fish fried	1
5592	Flingor fiber	Breakfast cereals bran flakes	Flingor - frukostflingor	Breakfast cereals	1
5589	Flingor kuddar	Breakfast cereals squares	Flingor - frukostflingor	Breakfast cereals	1
5593	Flingor majs	Breakfast cereals corn flakes	Flingor - frukostflingor	Breakfast cereals	1
5586	Flingor ringar	Breakfast cereals rings	Flingor - frukostflingor	Breakfast cereals	1
5594	Flingor ris	Breakfast cereals Special K	Flingor - frukostflingor	Breakfast cereals	1
727	Frukostflingor berik Frosties	Breakfast cereal fortified Frosties	Flingor - frukostflingor	Breakfast cereals	1
726	Frukostflingor berik Kalaspuffar	Breakfast cereal fortified Kalaspuffar	Flingor - frukostflingor	Breakfast cereals	1
713	Frukostflingor berik Rice Krispies	Breakfast cereal fortified Rice Krispies	Flingor - frukostflingor	Breakfast cereals	1
716	Frukostflingor ris socker kakao Coco Pops	Breakfast cereal rice sugar cocoa powder fortified Coco Pops	Flingor - frukostflingor	Breakfast cereals	1
5585	Granola start	Granola start	Flingor - frukostflingor	Breakfast cereals	1
5584	Müsli	Muesli	Flingor - frukostflingor	Breakfast cereals	1

[a] Foods in validation study and main survey Riksmaten ungdom 2016-17 = 1; Foods in main survey, only = 2; Foods in validation study, only = 3

Number	Food item (Swedish)	Food item	Main food group (Swedish)	Main food group	Study[a]
2470	Flytande margarin fett 80% berikad typ Mjuka Milda	Margarine liquid fat 80% fortified Milda	Flytande matfettsblandning	Fat of mixed origin >=80%fat	1
2730	Smör- & rapsolja	Butter and rapeseed oil liquid	Flytande matfettsblandning	Fat of mixed origin >=80%fat	1
550	Ananas	Pineapple	Frukt färsk fryst	Fruit fresh frozen	1
551	Apelsin	Orange	Frukt färsk fryst	Fruit fresh frozen	1
552	Aprikos	Apricot	Frukt färsk fryst	Fruit fresh frozen	1
553	Banan	Banana	Frukt färsk fryst	Fruit fresh frozen	1
559	Citron	Lemon	Frukt färsk fryst	Fruit fresh frozen	1
596	Citrusfrukter	Citrus fruits	Frukt färsk fryst	Fruit fresh frozen	1
2571	Exotisk frukt	Exotic fruit	Frukt färsk fryst	Fruit fresh frozen	1
561	Fikon	Figs	Frukt färsk fryst	Fruit fresh frozen	1
595	Frukt färsk	Fruit fresh	Frukt färsk fryst	Fruit fresh frozen	1
638	Fruktsallad	Fruit salad	Frukt färsk fryst	Fruit fresh frozen	1
520	Granatäpple	Pomegranate	Frukt färsk fryst	Fruit fresh frozen	1
521	Grapefrukt	Grapefruit	Frukt färsk fryst	Fruit fresh frozen	1
565	Kiwi grön	Kiwi fruit green	Frukt färsk fryst	Fruit fresh frozen	1
572	Lime	Lime	Frukt färsk fryst	Fruit fresh frozen	1
574	Mango	Mango	Frukt färsk fryst	Fruit fresh frozen	1
2593	Matbanan, friterad eller stekt	Plantains fried or deep fried	Frukt färsk fryst	Fruit fresh frozen	1
5739	Melon	Melon	Frukt färsk fryst	Fruit fresh frozen	1
579	Passionsfrukt	Passion fruit	Frukt färsk fryst	Fruit fresh frozen	1
580	Persika nektarin	Peach	Frukt färsk fryst	Fruit fresh frozen	1
582	Plommon	Plums	Frukt färsk fryst	Fruit fresh frozen	1
583	Päron	Pear	Frukt färsk fryst	Fruit fresh frozen	1
581	Sharon	Sharon	Frukt färsk fryst	Fruit fresh frozen	1
3855	Smoothie frukt och bär utan mjölk	Smoothie w/ fruits and berries	Frukt färsk fryst	Fruit fresh frozen	1
560	Småcitrus clementiner mandariner tangeriner satsumas	Small citrus fruit clementines mandarins satsumas	Frukt färsk fryst	Fruit fresh frozen	1
571	Sötkörsbär	Sweet cherries	Frukt färsk fryst	Fruit fresh frozen	1
587	Vindruvor	Grapes	Frukt färsk fryst	Fruit fresh frozen	1
588	Äpple m skal	Apple w/ skin	Frukt färsk fryst	Fruit fresh frozen	1
614	Ananas konserv m juice	Pineapple canned in juice	Frukt o bär konserverade	Fruit and berries preserved	1
636	Frukt konserverad	Fruits canned	Frukt o bär konserverade	Fruit and berries preserved	1
599	Aprikoser torkade	Apricots dried	Frukt o bär torkade	Fruit and berries dried	1
603	Dadlar torkade	Dates dried	Frukt o bär torkade	Fruit and berries dried	1
604	Fikon torkade	Figs dried	Frukt o bär torkade	Fruit and berries dried	1
4026	Frukt torkad och sötad	Fruit dried and sweetened	Frukt o bär torkade	Fruit and berries dried	1
605	Katrinplommon 32,5 % vatten	Prunes 32.5% moisture	Frukt o bär torkade	Fruit and berries dried	1
610	Russin	Raisins	Frukt o bär torkade	Fruit and berries dried	1
4049	Tranbär torkade	Cranberries dried	Frukt o bär torkade	Fruit and berries dried	1

[a] Foods in validation study and main survey Riksmaten ungdom 2016-17 = 1; Foods in main survey, only = 2; Foods in validation study, only = 3

Number	Food item (Swedish)	Food item	Main food group (Swedish)	Main food group	Study[a]
1881	Energibar choklad nötter Start	Energy bar chocolate nuts Start	Frukt o nötblandningar bars	Fruit and nuts mixes bars	1
3059	Sesamkakor sesamkex	Sesame biscuits	Frukt o nötblandningar bars	Fruit and nuts mixes bars	1
643	Apelsinjuice drickf	Orange juice RTD	Fruktjuice mm	Fruit juices etc	1
645	Citronjuice färskpressad	Lemon juice fresh	Fruktjuice mm	Fruit juices etc	1
649	Grapefruktjuice färskpressad	Grapefruit juice fresh	Fruktjuice mm	Fruit juices etc	1
662	Juice ospec	Juice unspec.	Fruktjuice mm	Fruit juices etc	1
652	Limejuice färskpressad	Lime juice fresh	Fruktjuice mm	Fruit juices etc	1
5859	Tropisk juice drickf	Tropical juice RTD	Fruktjuice mm	Fruit juices etc	1
659	Äppeljuice drickf	Apple juice RTD	Fruktjuice mm	Fruit juices etc	1
1152	Anka	Duck roasted	Fågel	Poultry	1
3939	Fågel vildfågel stekt	Game bird boiled	Fågel	Poultry	1
1191	Kalkon rökt	Smoked turkey	Fågel	Poultry	1
1190	Kyckling kött kokt stekt grillad ospec	Chicken hen boiled grilled fried	Fågel	Poultry	1
5902	Fajitas kyckling	Fajitas chicken	Fågelprodukter fågelrätter	Poultry products and dishes	1
2096	Flygande Jakob Kyckling bacon jordnötter banan	Chicken au gratin w/ bananas bacon and peanuts (Flygande Jacob)	Fågelprodukter fågelrätter	Poultry products and dishes	1
1207	Kyckling friterad Chicken McNuggets	Chicken deep-fried Chicken McNuggets	Fågelprodukter fågelrätter	Poultry products and dishes	1
5875	Kyckling m marinad tillagad typ Pulled chicken	Pulled chicken roasted	Fågelprodukter fågelrätter	Poultry products and dishes	1
5509	Kyckling Marengo	Chicken stew w/ tomato	Fågelprodukter fågelrätter	Poultry products and dishes	1
5932	Quesadillas	Quesadillas	Fågelprodukter fågelrätter	Poultry products and dishes	1
5694	Cottage cheese mellanmål typ Keso	Cottage cheese w/ fruits and nuts	Färskost o kvarg	Fresh cheese and quark	1
5692	Cottage cheese naturell typ Keso	Cottage cheese plain	Färskost o kvarg	Fresh cheese and quark	1
5693	Cottage cheese smaksatt typ Keso	Cottage cheese flavoured	Färskost o kvarg	Fresh cheese and quark	1
5697	Drickkvarg	Drinking quark	Färskost o kvarg	Fresh cheese and quark	1
99	Fetaost fett 25%	Feta cheese 25% fat	Färskost o kvarg	Fresh cheese and quark	1
109	Färskost extra light fett 5% typ Philadelphia	Cream cheese extra lgt 5% fat Philadelphia	Färskost o kvarg	Fresh cheese and quark	1
107	Färskost fett 27% typ Philadelphia	Cream cheese fat 27% Philadelphia	Färskost o kvarg	Fresh cheese and quark	1
5695	Kvarg naturell	Quark plain	Färskost o kvarg	Fresh cheese and quark	1
5696	Kvarg smaksatt	Quark flavoured	Färskost o kvarg	Fresh cheese and quark	1
2255	Mozzarella ost fett 16 %	Mozzarella cheese fat 20%	Färskost o kvarg	Fresh cheese and quark	1
5569	Glass Ben och Jerrys	Ice cream Ben & Jerrys	Glass	Ice cream	1
3127	Glass grädd	Ice cream mix	Glass	Ice cream	1
3128	Glass i bägare	Ice cream in cup	Glass	Ice cream	1
1695	Glass m chokladkex GB Sandwich	Ice cream in chocolate wafers GB Sandwich	Glass	Ice cream	1
5560	Glass mjölkfri	Milk free Ice Cream	Glass	Ice cream	1
5557	Glass yoghurtglass	Ice cream yoghurt ice cream	Glass	Ice cream	1

[a] Foods in validation study and main survey Riksmaten ungdom 2016-17 = 1; Foods in main survey, only = 2; Foods in validation study, only = 3

Number	Food item (Swedish)	Food item	Main food group (Swedish)	Main food group	Study[a]
3129	Glassbars	Ice cream bars	Glass	Ice cream	1
5553	Glasspinne liten	Ice lolly small	Glass	Ice cream	1
5554	Glasspinne stor	Ice lolly big	Glass	Ice cream	1
5561	Glasstrut kulglass	Ice cream cone	Glass	Ice cream	1
5556	Glasstrut liten	Ice cream cone small	Glass	Ice cream	1
5555	Glasstrut stor	Ice cream cone big	Glass	Ice cream	1
1705	Glasstårta fett 17% typ Vienetta	Ice cream cake fat 17%	Glass	Ice cream	1
5852	Glasstårta rund	Ice cream cake round	Glass	Ice cream	1
5570	Isglass ospec	Ice lolly unspec.	Glass	Ice cream	1
1703	Mjukglass bägare	Soft ice cream vanilla	Glass	Ice cream	1
3130	Mjukglass i strut	Soft ice cream in cone	Glass	Ice cream	1
1844	Sorbet	Sorbet	Glass	Ice cream	1
2964	Godisremmar	Candy straps cane	Godis ej choklad	Sweets excluding chocolate	1
2963	Klubba	Lollipop	Godis ej choklad	Sweets excluding chocolate	1
5604	Tablettask	Bulk confectionary in small box	Godis ej choklad	Sweets excluding chocolate	1
1876	Kexchoklad	Chocolate bar filled wafers covered w/ milk chocolate	Godis som innehåller choklad	Sweets miscellaneous	1
5601	Lösgodis påsgodis	Bulk confectionary	Godis som innehåller choklad	Sweets miscellaneous	1
2072	Müslibar berikad typ Special K Chocolate	Cereal bar enriched e.g. Special K Bar Chocolate	Godis som innehåller choklad	Sweets miscellaneous	1
5606	Naturgodis	Nature candy	Godis som innehåller choklad	Sweets miscellaneous	1
1860	Nöt- och chokladkräm Nutella	Chocolate hazelnut spread Nutella	Godis som innehåller choklad	Sweets miscellaneous	1
5763	Crème fraiche	Creme fraiche	Grädde creme fraiche	Creme, creme fraiche	1
1713	Gräddfil fett 12%	Sour cream fat 12%	Grädde creme fraiche	Creme, creme fraiche	1
5777	Matlagningsbas soja havre	Alternative cream from oats or soy beans	Grädde creme fraiche	Creme, creme fraiche	1
1717	Matlagningsgrädde fett 15%	Cooking cream fat 15%	Grädde creme fraiche	Creme, creme fraiche	1
1715	Vispgrädde fett 40%	Whipped cream fat 40%	Grädde creme fraiche	Creme, creme fraiche	1
470	Aubergine stekt	Aubergine fried	Grönsaker	Vegetables excluding potatoes	1
320	Avokado	Avocado	Grönsaker	Vegetables excluding potatoes	1
322	Blomkål	Cauliflower	Grönsaker	Vegetables excluding potatoes	1
2605	Blomkål stekt eller wokad	Cauliflower pan-fried or stir-fried	Grönsaker	Vegetables excluding potatoes	1
4939	Broccoli kokt	Broccoli boiled	Grönsaker	Vegetables excluding potatoes	1
4196	Brysselkål stekt eller wokad	Brussels sprouts fried or woked	Grönsaker	Vegetables excluding potatoes	1

[a] Foods in validation study and main survey Riksmaten ungdom 2016-17 = 1; Foods in main survey, only = 2; Foods in validation study, only = 3

Number	Food item (Swedish)	Food item	Main food group (Swedish)	Main food group	Study[a]
336	Fänkål	Fennel	Grönsaker	Vegetables excluding potatoes	1
2607	Fänkål stekt eller wokad	Fennel pan-fried or stir-fried	Grönsaker	Vegetables excluding potatoes	1
337	Grönkål	Kale	Grönsaker	Vegetables excluding potatoes	1
3952	Grönsaker syltade	Vegetables brawned	Grönsaker	Vegetables excluding potatoes	1
3927	Grönsallat gurka tomat majs utan dressing	Lettuce mixed cucumber tomato corn w/ out dressing	Grönsaker	Vegetables excluding potatoes	1
444	Grönsallat gurka tomat paprika u dressing	Salad lettuce mix w/ cucumber tomato sweet pepper w/o dressing	Grönsaker	Vegetables excluding potatoes	1
390	Grönsallat gurka tomat u dressing	Lettuce mixed cucumber tomato w/o dressing	Grönsaker	Vegetables excluding potatoes	1
5759	Guacamole	Guacamole	Grönsaker	Vegetables excluding potatoes	1
339	Gurka	Cucumber	Grönsaker	Vegetables excluding potatoes	1
487	Gurka inlagd	Cucumber pickled	Grönsaker	Vegetables excluding potatoes	1
340	Isbergssallat	Iceberg lettuce	Grönsaker	Vegetables excluding potatoes	1
492	Kronärtskocka kokt	Globe artichoke boiled	Grönsaker	Vegetables excluding potatoes	1
344	Lök gul	Onions yellow	Grönsaker	Vegetables excluding potatoes	1
446	Lök stekt	Onion fried	Grönsaker	Vegetables excluding potatoes	1
400	Majskorn konserv u lag	Corn kernels canned drained	Grönsaker	Vegetables excluding potatoes	1
4154	Majskorn stekta wokade	Corn fried	Grönsaker	Vegetables excluding potatoes	1
401	Oliver gröna m paprikafyllning avrunna	Olives green marinated drained	Grönsaker	Vegetables excluding potatoes	1
392	Paprika grön gul röd	Sweet peppers green yellow red	Grönsaker	Vegetables excluding potatoes	1
2610	Paprika stekt grillad eller wokad	Sweet/bell pepper grilled, pan-fried or stir-fried	Grönsaker	Vegetables excluding potatoes	1
2611	Pumpa eller squash, stekt eller wokad	Pumpkin or marrow pan-fried or stir-fried	Grönsaker	Vegetables excluding potatoes	1

[a] Foods in validation study and main survey Riksmaten ungdom 2016-17 = 1; Foods in main survey, only = 2; Foods in validation study, only = 3

Number	Food item (Swedish)	Food item	Main food group (Swedish)	Main food group	Study[a]
354	Purjolök	Leek	Grönsaker	Vegetables excluding potatoes	1
2612	Purjolök stekt eller wokad	Leek pan-fried or stir-fried	Grönsaker	Vegetables excluding potatoes	1
355	Rödkål	Red cabbage	Grönsaker	Vegetables excluding potatoes	1
358	Salladskål	Chinese cabbage	Grönsaker	Vegetables excluding potatoes	1
2639	Sallat, olika sorter	Salad, various kinds	Grönsaker	Vegetables excluding potatoes	1
3867	Skott	Sprouts	Grönsaker	Vegetables excluding potatoes	1
3959	Sparris stekt eller wokad	Asparagus fried or woked	Grönsaker	Vegetables excluding potatoes	1
455	Spenat fräst	Spinach fried w/o browning	Grönsaker	Vegetables excluding potatoes	1
5743	Spenat Mangold	Spinach Mangold	Grönsaker	Vegetables excluding potatoes	1
362	Squash	Summer squash	Grönsaker	Vegetables excluding potatoes	1
321	Stjälkselleri	Celery	Grönsaker	Vegetables excluding potatoes	1
406	Surkål konserv m lag	Sauerkraut canned whole contents	Grönsaker	Vegetables excluding potatoes	1
364	Tomat	Tomato	Grönsaker	Vegetables excluding potatoes	1
4240	Tomat stekt eller wokad	Tomato fried	Grönsaker	Vegetables excluding potatoes	1
422	Tomater krossade konserv m lag	Chopped tomatoes canned whole contents	Grönsaker	Vegetables excluding potatoes	1
370	Vitkål	Cabbage	Grönsaker	Vegetables excluding potatoes	1
2617	Vitkål stekt eller wokad	White cabbage pan-fried or stir-fried	Grönsaker	Vegetables excluding potatoes	1
5744	Örtkryddor	Herbs	Grönsaker	Vegetables excluding potatoes	1
417	Ajvar relish grönsakspasta m paprika aubergine	Ajvar relish vegetable paste w/ sweet peppers and aubergine	Grönsaks- rotfrukts- baljväxträtter o produkter	Vegetables leguminous plant root vegetables dishes and products	1

[a] Foods in validation study and main survey Riksmaten ungdom 2016-17 = 1; Foods in main survey, only = 2; Foods in validation study, only = 3

Number	Food item (Swedish)	Food item	Main food group (Swedish)	Main food group	Study[a]
2887	Auberginеröra	Aubergine mix	Grönsaks- rotfrukts- baljväxträtter o produkter	Vegetables leguminous plant root vegetables dishes and products	1
488	Bostongurka	Cucumber mix pickled	Grönsaks- rotfrukts- baljväxträtter o produkter	Vegetables leguminous plant root vegetables dishes and products	1
3820	Bruna bönor rullpack	Swedish brown beans roll	Grönsaks- rotfrukts- baljväxträtter o produkter	Vegetables leguminous plant root vegetables dishes and products	1
5928	Burrito	Burrito	Grönsaks- rotfrukts- baljväxträtter o produkter	Vegetables leguminous plant root vegetables dishes and products	1
2662	Bönor marinerade	Marinated beans	Grönsaks- rotfrukts- baljväxträtter o produkter	Vegetables leguminous plant root vegetables dishes and products	1
458	Champinjonstuvning	Mushrooms in thick white sauce	Grönsaks- rotfrukts- baljväxträtter o produkter	Vegetables leguminous plant root vegetables dishes and products	1
2064	Falafel kikärtskroketter frysta	Falafel chickpea-croquettes frozen	Grönsaks- rotfrukts- baljväxträtter o produkter	Vegetables leguminous plant root vegetables dishes and products	1
2563	Friterade lökringar	Onion rings deep fried	Grönsaks- rotfrukts- baljväxträtter o produkter	Vegetables leguminous plant root vegetables dishes and products	1
502	Grönsaker blandade fermenterade	Vegetables mixed fermented	Grönsaks- rotfrukts- baljväxträtter o produkter	Vegetables leguminous plant root vegetables dishes and products	1
4001	Grönsaker ospec. friterade	Vegetables unspec. deep fried	Grönsaks- rotfrukts- baljväxträtter o produkter	Vegetables leguminous plant root vegetables dishes and products	1
476	Grönsaker stuvade ospec.	Vegetables in thick white sauce	Grönsaks- rotfrukts- baljväxträtter o produkter	Vegetables leguminous plant root vegetables dishes and products	1
439	Grönsaksbiff stekt	Vegetable patties fried	Grönsaks- rotfrukts- baljväxträtter o produkter	Vegetables leguminous plant root vegetables dishes and products	1
5500	Grönsaksgratäng	Vegetable gratin	Grönsaks- rotfrukts- baljväxträtter o produkter	Vegetables leguminous plant root vegetables dishes and products	1

[a] Foods in validation study and main survey Riksmaten ungdom 2016-17 = 1; Foods in main survey, only = 2; Foods in validation study, only = 3

Number	Food item (Swedish)	Food item	Main food group (Swedish)	Main food group	Study[a]
4018	Grönsakspaté	Vegetable paté	Grönsaks- rotfrukts- baljväxträtter o produkter	Vegetables leguminous plant root vegetables dishes and products	1
3051	Hummus - kikärtsröra	Hummus dip/spread made from chickpeas	Grönsaks- rotfrukts- baljväxträtter o produkter	Vegetables leguminous plant root vegetables dishes and products	1
2701	Kikärtor marinerade	Marinated chickpeas	Grönsaks- rotfrukts- baljväxträtter o produkter	Vegetables leguminous plant root vegetables dishes and products	1
478	Kikärtsgryta grönsaksgryta	Vegetable casserole unspec.	Grönsaks- rotfrukts- baljväxträtter o produkter	Vegetables leguminous plant root vegetables dishes and products	1
2700	Linser marinerade	Marinated lentils	Grönsaks- rotfrukts- baljväxträtter o produkter	Vegetables leguminous plant root vegetables dishes and products	1
2638	Linsgryta böngryta	Vegetarian casserole w/ beans and lentils	Grönsaks- rotfrukts- baljväxträtter o produkter	Vegetables leguminous plant root vegetables dishes and products	1
2083	Ratatouille	Ratatouille	Grönsaks- rotfrukts- baljväxträtter o produkter	Vegetables leguminous plant root vegetables dishes and products	1
2058	Rostad lök	Roasted onion	Grönsaks- rotfrukts- baljväxträtter o produkter	Vegetables leguminous plant root vegetables dishes and products	1
5510	Rotfruktsgratäng	Root vegetable gratin	Grönsaks- rotfrukts- baljväxträtter o produkter	Vegetables leguminous plant root vegetables dishes and products	1
302	Rotmos hemlagad	Mashed swedes and potatoes home-made	Grönsaks- rotfrukts- baljväxträtter o produkter	Vegetables leguminous plant root vegetables dishes and products	1
3858	Råkostsallad med morot vitkål etc	Vegetable salad w/ carrot cabbage etc	Grönsaks- rotfrukts- baljväxträtter o produkter	Vegetables leguminous plant root vegetables dishes and products	1
453	Rödkål tillagad	Cabbage red and apples fried boiled	Grönsaks- rotfrukts- baljväxträtter o produkter	Vegetables leguminous plant root vegetables dishes and products	1
365	Soltorkade tomater i olja	Sundried tomatoes marinated in oil	Grönsaks- rotfrukts- baljväxträtter o produkter	Vegetables leguminous plant root vegetables dishes and products	1

[a] Foods in validation study and main survey Riksmaten ungdom 2016-17 = 1; Foods in main survey, only = 2; Foods in validation study, only = 3

Number	Food item (Swedish)	Food item	Main food group (Swedish)	Main food group	Study[a]
824	Vinbladsdolma m ris konserv	Wine leaves stuffed w/ rice canned	Grönsaks- rotfrukts- baljväxträtter o produkter	Vegetables leguminous plant root vegetables dishes and products	1
901	Vita bönor i tomatsås konserv	White beans baked in tomato sauce canned	Grönsaks- rotfrukts- baljväxträtter o produkter	Vegetables leguminous plant root vegetables dishes and products	1
467	Vitkålssallad pizzasallad	Cabbage salad Swedish pizza salad	Grönsaks- rotfrukts- baljväxträtter o produkter	Vegetables leguminous plant root vegetables dishes and products	1
2718	Vitlök marinerad	Marinated garlic	Grönsaks- rotfrukts- baljväxträtter o produkter	Vegetables leguminous plant root vegetables dishes and products	1
3859	Grönsaker rotfrukter stekta wokade	Mixed vegetables and root crops fried	Grönsaksblandningar med rotfrukter och eller baljväxter	Vegetable mixes w root vegetables and or leguminous plants	1
425	Wokgrönsaker Classic wokade i rapsolja	Stir-fry vegetable mix Classic woked in rapeseed oil	Grönsaksblandningar med rotfrukter och eller baljväxter	Vege	1
471	Ärtor morötter tillagade	Peas and carrots boiled	Grönsaksblandningar med rotfrukter och eller baljväxter	Vegetable mixes w root vegetables and or leguminous plants	1
427	Grönsaksjuice konserv el pastöriserad drickf	Vegetable juice canned or pasteurised RTD	Grönsaksjuice rotfruktsjuice	Vegetable juice	1
301	Morotsjuice	Carrot juice	Grönsaksjuice rotfruktsjuice	Vegetable juice	1
408	Tomatjuice konserv drickf	Tomato juice canned RTD	Grönsaksjuice rotfruktsjuice	Vegetable juice	1
3877	Gröt av blandade gryn	Porridge multigrain	Gröt	Porridge	1
677	Havregrynsgröt fullkorn	Oatmeal porridge wholemeal	Gröt	Porridge	1
2810	Mannafrutti	Rice porridge e.g. Risifrutti Manna	Gröt	Porridge	1
682	Mannagrynsgröt	Semolina porridge	Gröt	Porridge	1
655	Risgrynsgröt	Rice porridge	Gröt	Porridge	1
3272	Risifrutti original	Rice pudding w/ fruit sauce e.g. Risifrutti original	Gröt	Porridge	1
776	Fiskburgare McDonalds	Fish burger Mac Donalds	Hamburgare med bröd (kött, fisk, fågel, vegetarisk)	Hamburger with bread	1
5838	Hamburgare big mac, whopper, frisco	Hamburger big mac whopper frisco	Hamburgare med bröd (kött, fisk, fågel, vegetarisk)	Hamburger with bread	1
5840	Hamburgare liten	Hamburger small	Hamburgare med bröd (kött, fisk, fågel, vegetarisk)	Hamburger with bread	1
5839	Hamburgare stor, Big tasty, super cheese n bacon	Hamburger large big tasty super cheese n bacon	Hamburgare med bröd (kött, fisk, fågel, vegetarisk)	Hamburger with bread	1
5841	Hamburgare utan bröd	Hamburger w/o bread	Hamburgare med bröd (kött, fisk, fågel, vegetarisk)	Hamburger with bread	1

[a] Foods in validation study and main survey Riksmaten ungdom 2016-17 = 1; Foods in main survey, only = 2; Foods in validation study, only = 3

Number	Food item (Swedish)	Food item	Main food group (Swedish)	Main food group	Study[a]
775	Kycklingburgare McDonalds	Chicken burger Mac Donalds	Hamburgare med bröd (kött, fisk, fågel, vegetarisk)	Hamburger with bread	1
3266	Vegetarisk burgare med bröd sallad dressing	Vegetarian burger w/ bread salad sauce	Hamburgare med bröd (kött, fisk, fågel, vegetarisk)	Hamburger with bread	1
5701	Lättmargarin fett 40-60%	Margarine fat 40-60%	Hård matfettsblandning	Mixed origin fat	1
5700	Margarin fett 60-80%	Margarine fat 60-80%	Hård matfettsblandning	Mixed origin fat	1
5699	Matfettsblandning	Butter fat 60-80 %	Hård matfettsblandning	Mixed origin fat	1
95	Ost hårdost fett 17%	Hard cheese fat 17%	Hård ost mm	Hard cheese	1
5778	Ost hårdost fett 27%	Cheese fat 27%	Hård ost mm	Hard cheese	1
103	Parmesanost hårdost fett 30%	Parmesan hard cheese grated 30% fat	Hård ost mm	Hard cheese	1
1604	Fullkornsskorpor osötade	Wheat wholemeal rusks	Hårt bröd	Crisp bread	1
3252	Grissini	Grissini Italian breadsticks	Hårt bröd	Crisp bread	1
5562	Hårt bröd grovt	Crisp bread wholegrain	Hårt bröd	Crisp bread	1
5597	Hårt bröd tunt	Crisp bread thin	Hårt bröd	Crisp bread	1
5598	Hårt bröd vitt	Crisp bread white	Hårt bröd	Crisp bread	1
215	Hårt tunnbröd vete korn socker fiber ca 3% Wasa Tunnbröd	Crisp bread thin wheat barley sugar fibre c 3% Wasa Tunnbröd	Hårt bröd	Crisp bread	1
5794	Inälvs- organmat ej lever¨	Offals	Inälvor och organ	Offals	1
1484	Lever stekt	Liver pan fried	Inälvor organ produkter o rätter	Offal products and dishes	1
1479	Leverbiff mald lever stekt hemlagad	Liver patties fried home-made	Inälvor organ produkter o rätter	Offal products and dishes	1
4038	Levergryta ospec.	Liver casserole unspec.	Inälvor organ produkter o rätter	Offal products and dishes	1
1541	Pölsa värmd stekt	Hash made w/ eg barley offal heated fried	Inälvor organ produkter o rätter	Offal products and dishes	1
1957	Kaffe bryggt	Coffee brewed	Kaffe	Coffee infusion	1
2816	Caffè latte	Caffè latte	Kaffe te med mjölk	Coffee Tea with milk	1
4057	Chai latte	Chai latte	Kaffe te med mjölk	Coffee Tea with milk	1
1529	Falukorv stekt	Sausage ('Falu' sausage) pan-fried	Korv	Sausages	1
1531	Korv fermenterad kött 59% stekt typ isterband	Sausage fermented meat fried 59% e.g. Småländska isterband	Korv	Sausages	1
1491	Korv kött 48% kokt typ fläskkorv	Sausage meat 48% boiled e.g. fläskkorv	Korv	Sausages	1
1513	Korv kött 73% stekt typ Chorizo	Sausage meat 73% fried e.g. Chorizo	Korv	Sausages	1
1542	Korvpålägg ospec	Sausage for sandwich unspec.	Korv	Sausages	1
2097	Prinskorv stekt	Miniature frankfurter('Prince' sausage) pan-fried	Korv	Sausages	1
1498	Påläggskorv salami fett 34-44%	Salami sausage fat 33% smoked	Korv	Sausages	1
3740	Varmkorv grillkorv wienerkorv av kyckling kalkon eller struts kokt	Hot dog frankfurter made of chicken turkey or ostrich boiled	Korv	Sausages	1
1535	Varmkorv stekt	Sausage hot dog fried	Korv	Sausages	1
2853	Korv Stroganoff m grädde	Sausage Stroganoff w/ cooking cream	Korvrätter	Sausage diches	1
5784	Korvgryta m korv chorizo grönsaker rotfrukter	Sausage casserole w/ sausage chorizo vegetables root vegetables	Korvrätter	Sausage diches	1

[a] Foods in validation study and main survey Riksmaten ungdom 2016-17 = 1; Foods in main survey, only = 2; Foods in validation study, only = 3

Number	Food item (Swedish)	Food item	Main food group (Swedish)	Main food group	Study[a]
3249	Bars måltidsersättning typ Nutrilett	Meal replacement bar e.g. Nutrilett	Kost- o näringspreparat	Diet products, nutritional therapy	1
3123	Måltidsersättning drickfärdig	Meal replacment RTD e.g. Nutrilett	Kost- o näringspreparat	Diet products, nutritional therapy	1
3250	Proteinbar	Proteinbar	Kost- o näringspreparat	Diet products, nutritional therapy	1
3550	Proteintillskott i pulverform Whey-80	Protein supplement powder	Kost- o näringspreparat	Diet products, nutritional therapy	1
3563	Återhämtningsprodukt Gainer-Pro pulver	Recovery product Gainer-Pro powder	Kost- o näringspreparat	Diet products, nutritional therapy	1
2306	Kanel	Cinnamon	Kryddor	Spices	1
5798	Biff tillagad	Steak pan fried	Kött färskt fryst tillagat	Red meat fresh frozen cooked	1
4174	Carpaccio nötkött	Beef carpaccio	Kött färskt fryst tillagat	Red meat fresh frozen cooked	1
5797	Filé tillagad	Loin pan fried	Kött färskt fryst tillagat	Red meat fresh frozen cooked	1
1045	Gris revbensspjäll ugnsstekt grillat	Spare ribs pork barbecued	Kött färskt fryst tillagat	Red meat fresh frozen cooked	1
5796	Kotlett karré tillagad	Chops loin collar chaps pan fried	Kött färskt fryst tillagat	Red meat fresh frozen cooked	1
5799	Kött grillat	Meat grilled	Kött färskt fryst tillagat	Red meat fresh frozen cooked	1
3988	Kött ospec. kokt	Meat unspec. boiled	Kött färskt fryst tillagat	Red meat fresh frozen cooked	1
4183	Köttfärs ospec. stekt	Minced meat unspec. Fried	Kött färskt fryst tillagat	Red meat fresh frozen cooked	1
2908	Lammrevbensspjäll	Lamb racks	Kött färskt fryst tillagat	Red meat fresh frozen cooked	1
2826	Lövbiff stekt	Top round steak fried (thin slices)	Kött färskt fryst tillagat	Red meat fresh frozen cooked	1
1017	Nöt entrecôte stekt	Beef cube roll fried	Kött färskt fryst tillagat	Red meat fresh frozen cooked	1
5853	Pulled beef	Pulled beef	Kött färskt fryst tillagat	Red meat fresh frozen cooked	1
2832	Skav stekt	Meat thin slices pan-fried	Kött färskt fryst tillagat	Red meat fresh frozen cooked	1
2829	Stek tillagad	Roasted steak	Kött färskt fryst tillagat	Red meat fresh frozen cooked	1
1004	Gris bacon stekt	Bacon pork fried	Kött processat	Processed meat	1

[a] Foods in validation study and main survey Riksmaten ungdom 2016-17 = 1; Foods in main survey, only = 2; Foods in validation study, only = 3

Number	Food item (Swedish)	Food item	Main food group (Swedish)	Main food group	Study[a]
1016	Gris kassler kotlettrad rökt stekt el värmd	Pork loin smoked fried or heated	Kött processat	Processed meat	1
1020	Gris sidfläsk rimmat stekt	Cured pork belly fried	Kött processat	Processed meat	1
1010	Gris skinka fett 1-3% skivad rökt	Smoked ham pork fat 1-3% sliced	Kött processat	Processed meat	1
1008	Gris skinka italiensk lufttorkad	Italian prosciutto cured air-dried ham pork	Kött processat	Processed meat	1
1015	Gris skinka julskinka fett ca 4,5% rimmad kokt	Cured ham Christmas ham pork fat 4.5% boiled	Kött processat	Processed meat	1
998	Häst kött hamburgerkött rökt	Horse meat smoked	Kött processat	Processed meat	1
3019	Kött rökt	Smoked meat	Kött processat	Processed meat	1
1475	Leverpastej skivbar fett 26%	Liver paste or paté slicable fat c 26%	Kött processat	Processed meat	1
999	Nöt saltkött rimmat kokt	Beef meat cured boiled	Kött processat	Processed meat	1
1002	Ren kött torkat	Reindeer meat dried	Kött processat	Processed meat	1
3155	Skinka kokt	Boiled ham	Kött processat	Processed meat	1
5809	Köttfärssås	Minced meat sauce	Köttprodukter kötträtter	Meat products meat dishes	1
898	Chili con carne	Chilli con carne	Köttprodukter kötträtter	Meat products meat dishes	1
5850	Dumplings m fläsk räkor	Dumplings w/ pork shrimp	Köttprodukter kötträtter	Meat products meat dishes	1
3606	Fläskkött friterat	Pork meat fried	Köttprodukter kötträtter	Meat products meat dishes	1
3115	Grillspett med kött och grönsaker	Skewer w/ meat and vegetables	Köttprodukter kötträtter	Meat products meat dishes	1
5847	Gris m marinad tillagad typ Pulled pork	Pulled pork roasted	Köttprodukter kötträtter	Meat products meat dishes	1
5808	Järpar stekta	Minced meat rolls pan fried	Köttprodukter kötträtter	Meat products meat dishes	1
2507	Kebab special m pommes frites	Kebab spec. w/ french fries	Köttprodukter kötträtter	Meat products meat dishes	1
5810	Kebabkött stekt	Döner kebab fried	Köttprodukter kötträtter	Meat products meat dishes	1
512	Kålpudding	Cabbage pudding	Köttprodukter kötträtter	Meat products meat dishes	1
5806	Köttbullar stekta	Meat balls pan fried	Köttprodukter kötträtter	Meat products meat dishes	1
2923	Köttfärsgratäng typ tacogratäng	Minced meat gratin e.g. taco gratin	Köttprodukter kötträtter	Meat products meat dishes	1
5805	Köttfärslimpa ugnsbakad	Meat loaf oven baked	Köttprodukter kötträtter	Meat products meat dishes	1
2844	Köttgryta nöt och gris	Beef stew w/ beef and pork	Köttprodukter kötträtter	Meat products meat dishes	1
1482	Lantpaté	Country paté	Köttprodukter kötträtter	Meat products meat dishes	1
252	Lapskojs	Lobscouse corned beef and mashed potatoes	Köttprodukter kötträtter	Meat products meat dishes	1
515	Moussaka ugnsbakad	Moussaka	Köttprodukter kötträtter	Meat products meat dishes	1
5929	Nachotallrik	Nacho plate	Köttprodukter kötträtter	Meat products meat dishes	1
5930	Nachotallrik stor	Nacho plate large	Köttprodukter kötträtter	Meat products meat dishes	1
1124	Nöt färs tacokryddad stekt	Minced meat filling for taco shells	Köttprodukter kötträtter	Meat products meat dishes	1
1043	Oxrulad nöt stekt	Beef rolls fried	Köttprodukter kötträtter	Meat products meat dishes	1
5807	Pannbiff stekt	Patty pan fried	Köttprodukter kötträtter	Meat products meat dishes	1
1081	Renskavsgryta	Reindeer thin slices casserole	Köttprodukter kötträtter	Meat products meat dishes	1
3993	Råbiff	Steak tartare	Köttprodukter kötträtter	Meat products meat dishes	1
250	Sjömansbiff hemlagad	Sailor's beef home-made	Köttprodukter kötträtter	Meat products meat dishes	1
1066	Wienerschnitzel gris	Wiener schnitzel pork	Köttprodukter kötträtter	Meat products meat dishes	1
1831	Cola light	Diet Cola sweetened w/ synthetic sweeteners	Lightdrycker u energi	Beverages without energy, light soft drinks etc.	1

[a] Foods in validation study and main survey Riksmaten ungdom 2016-17 = 1; Foods in main survey, only = 2; Foods in validation study, only = 3

Number	Food item (Swedish)	Food item	Main food group (Swedish)	Main food group	Study[a]
1830	Lightläsk	Carbonated soft drinks sweetened w/ synthetic sweeteners	Lightdrycker u energi	Beverages without energy, light soft drinks etc.	1
1834	Saft sötad m sötningsmedel drickf	Cordial artificial sweetener RTD	Lightdrycker u energi	Beverages without energy, light soft drinks etc.	1
1919	Likör söt vol. % 24	Liqueur sweet vol. % 24	Likör	Liqueur	1
51	Gurkmajonnäs el gurksallad gatukök	Cucumber mayonnaise or cucumber salad fast food	Majonnässallad röror	Mayonnaise salad	1
3852	Kycklingröra	Chicken in a mayonnaise/sour cream based sauce	Majonnässallad röror	Mayonnaise salad	1
55	Mimosasallad	Mimosa salad w/ fruit	Majonnässallad röror	Mayonnaise salad	1
52	Räkmajonnäs el räksallad gatukök	Shrimp mayonnaise or shrimp salad fast food	Majonnässallad röror	Mayonnaise salad	1
3871	Rödbetssallad	Beetroot salad mayonnaise	Majonnässallad röror	Mayonnaise salad	1
2107	Skagenröra	Shellfish and mayonnaise mix	Majonnässallad röror	Mayonnaise salad	1
3053	Tapenade olivröra	Tapenade	Majonnässallad röror	Mayonnaise salad	1
3874	Tonfiskröra	Tuna mayonnaise salad	Majonnässallad röror	Mayonnaise salad	1
2792	Bovete kokt med salt	Buckwheat w/ salt	Matgryn	Grain bulgur, couscous	1
5733	Bulgur kokt vanlig o fullkorn	Bulgur boiled regular and wholegrain	Matgryn	Grain bulgur, couscous	1
5735	Couscous kokt	Couscous boiled	Matgryn	Grain bulgur, couscous	1
835	Majsgryn polenta kokt	Corn flour polenta boiled	Matgryn	Grain bulgur, couscous	1
5734	Matgryn kokt	Grains boiled	Matgryn	Grain bulgur, couscous	1
3518	Quinoa röd kokt m salt	Quinoa red boiled w/ salt	Matgryn	Grain bulgur, couscous	1
66	Mesost fett ca 30% berik	Whey cheese fat 30% fortified	Mesvaror	Whey products	1
68	Messmör fett 5% berik	Whey spread fat 5% fortified	Mesvaror	Whey products	1
2822	Bagels	Bagel	Mjukt bröd	Soft bread	1
4215	Bröd friterat typ Langos (utan fyllning)	Bread deep fried e.g langos	Mjukt bröd	Soft bread	1
5566	Bröd halvljust	Semi whole grain bread	Mjukt bröd	Soft bread	1
5568	Bröd mörkt	Whole grain bread	Mjukt bröd	Soft bread	1
3791	Bröd vitt fibrer ca 5% typ Jättefranska	White bread fibre c 5% e.g. Jättefranska	Mjukt bröd	Soft bread	1
3247	Brödkrutonger	Croutons, plain	Mjukt bröd	Soft bread	1
229	Croissant fransk giffel	Croissant	Mjukt bröd	Soft bread	1
3818	Hamburgerbröd	Hamburger bread	Mjukt bröd	Soft bread	1
194	Korvbröd	Hot dog bun	Mjukt bröd	Soft bread	1
4067	Naan indiskt mjukt bröd	Indian naan bread	Mjukt bröd	Soft bread	1
5567	Polarbröd	Tea biscuit	Mjukt bröd	Soft bread	1
205	Scones vitt bröd vete	White bread wheat scones	Mjukt bröd	Soft bread	1
217	Tunnbröd vitt bröd mjukt mjölk fibrer ca 4%	Bread soft thin w/ milk fibre c 4%	Mjukt bröd	Soft bread	1
198	Vitt bröd osötat typ italienskt fibrer ca 2,5%	White bread e.g. Italian unsweetened fibre c 2.5%	Mjukt bröd	Soft bread	1
203	Vitt bröd tortilla fibrer 4,6%	White bread tortilla fibre 4.6%	Mjukt bröd	Soft bread	1
193	Vitt bröd vatten typ pitabröd fibrer ca 3,5%	White bread e.g. pita fibre c 3.5%	Mjukt bröd	Soft bread	1

[a] Foods in validation study and main survey Riksmaten ungdom 2016-17 = 1; Foods in main survey, only = 2; Foods in validation study, only = 3

Number	Food item (Swedish)	Food item	Main food group (Swedish)	Main food group	Study[a]
200	Vitt bröd vete baguette	Baguette white bread wheat	Mjukt bröd	Soft bread	1
5737	Havregryn	Oats rolled oats	Mjöl stärkelse kli	Flour starch bran	1
5736	Kli	Bran	Mjöl stärkelse kli	Flour starch bran	1
680	Rågkross fullkorn rågflingor ångprep	Rye crushed grains or flakes steamed wholemeal	Mjöl stärkelse kli	Flour starch bran	1
708	Vetegroddar	Wheat germs	Mjöl stärkelse kli	Flour starch bran	1
116	Kondenserad mjölk konserv konc sockrad fett ca 9%	Condensed milk sweetened canned fat ca 9%	Mjölk	Milk	1
5613	Lättmjölk	Milk low-fat	Mjölk	Milk	1
5614	Mellanmjölk	Milk medium-fat	Mjölk	Milk	1
5615	Mjölk fett >3%	Milk fat >3%	Mjölk	Milk	1
5873	Mjölkdryck extra protein 0,5% fett	Milk w/ extra protein 0.5% fat	Mjölk	Milk	1
118	Mjölkpulver fett 1%	Skimmed milk powder fat 1%	Mjölk	Milk	1
5874	Mjölkshake extra protein 1,5 % fett	Milk flavoured extra protein 1.5% fat	Mjölk	Milk	1
5831	Caramel frappuccino	Caramel frappuccino	Mjölkdryck chokladdryck milkshake smothie m yoghurt	Cocoa milkdrink milkshake smothie w youghurt	1
122	Milkshake choklad jordgubb	Milkshake chocolate strawberry	Mjölkdryck chokladdryck milkshake smothie m yoghurt	Cocoa milkdrink milkshake smothie w youghurt	1
160	Smoothie m bär yoghurt banan juice	Smoothie w/ berries yoghurt banana juice	Mjölkdryck chokladdryck milkshake smothie m yoghurt	Cocoa milkdrink milkshake smothie w youghurt	1
157	Varm choklad mjölk fett 1,5%	Cocoa w/ milk fat 1,5%	Mjölkdryck chokladdryck milkshake smothie m yoghurt	Cocoa milkdrink milkshake smothie w youghurt	1
2256	Yoghurtdryck fett 0,5 % Yoggi Yalla!	Yoghurt drink 0.5% fat e.g. Yoggi Yalla!	Mjölkdryck chokladdryck milkshake smothie m yoghurt	Cocoa milkdrink milkshake smothie w youghurt	1
2733	Grekisk eller turkisk yoghurt (10 % fett)	Greek or Turkish yoghurt 10% fat	Naturell fil yoghurt	Natural fermented products	1
5775	Matlagningsyoghurt	Yoghurt for cooking	Naturell fil yoghurt	Natural fermented products	1
3064	Brända mandlar	Almonds candied	Nötter frön	Nuts and seeds	1
3069	Chilinötter	Chili peanuts	Nötter frön	Nuts and seeds	1
5730	Frön	Seeds	Nötter frön	Nuts and seeds	1
1559	Jordnötssmör	Peanut butter	Nötter frön	Nuts and seeds	1
1562	Jordnötter rostade saltade	Peanuts roasted salted	Nötter frön	Nuts and seeds	1
1560	Jordnötter torkade	Peanuts dried	Nötter frön	Nuts and seeds	1
1564	Kokosflingor torkade	Shredded coconut dried	Nötter frön	Nuts and seeds	1
5729	Mandlar	Almond	Nötter frön	Nuts and seeds	1
3258	Nöt och frukt bärmix	Nuts and dried fruits berries	Nötter frön	Nuts and seeds	1
3140	Nötter blandade	Mixed nuts	Nötter frön	Nuts and seeds	1
3139	Nötter rostade saltade	Mixed nuts salted	Nötter frön	Nuts and seeds	1
1575	Sötmandel torkad	Almonds dried	Nötter frön	Nuts and seeds	1

[a] Foods in validation study and main survey Riksmaten ungdom 2016-17 = 1; Foods in main survey, only = 2; Foods in validation study, only = 3

Number	Food item (Swedish)	Food item	Main food group (Swedish)	Main food group	Study[a]
35	Olivolja	Olive oil	Olja	Vegetable oils	1
2189	Rapsolja	Rapeseed oil	Olja	Vegetable oils	1
3652	Ost sojabaserad hårdost	Hard cheese soy protein	Ost med vegetabiliskt fett	Cheese with vegetable fat	1
905	Tofu sojabönsost	Tofu soya bean curd	Ost med vegetabiliskt fett	Cheese with vegetable fat	1
2247	Tofutti (creamy smooth) original	Tofutti (creamy smooth) original	Ost med vegetabiliskt fett	Cheese with vegetable fat	1
110	Camembert vitmögelost friterad	Camembert cheese white mould ripened deep-fried	Osträtter	Cheese dish	1
2564	Chili cheese	Chili cheese	Osträtter	Cheese dish	1
2899	Halloumi stekt eller grillad	Halloumi cheese fried or grilled	Osträtter	Cheese dish	1
748	Crêpes pannkaka tunn fylld ospec.	Pancake thin filled unspec.	Pannkakor, våfflor, crêpes	Panncake,	1
731	Pannkaka tunn hemlagad	Pancakes thin home-made	Pannkakor, våfflor, crêpes	Panncake,	1
5783	Ugnspannkaka o fläskpannkaka	Pancake thick pancake w/ fried pork	Pannkakor, våfflor, crêpes	Panncake,	1
736	Våfflor äggvåfflor	Waffles plain or egg waffles	Pannkakor, våfflor, crêpes	Panncake,	1
5738	Nudlar kokta	Noodles boiled	Pasta	pasta	1
3828	Pasta >50% fullkorn kokt m salt	Pasta whole grain >50% boiled w/ salt	Pasta	pasta	1
3756	Pasta kokt m salt	Pasta boiled w/ salt	Pasta	pasta	1
2542	Lasagne nötfärs eller blandfärs	Lasagna w/ minced beef or beef and pork mince	Pastarätter	Pasta dishes	1
866	Lasagne vegetarisk spenat chèvre	Lasagne veg.	Pastarätter	Pasta dishes	1
4160	Nudelwok med kött grönsaker	Stir-fried noodles w/ meat, chicken or sausage	Pastarätter	Pasta dishes	1
5804	Pasta carbonara	Pasta carbonara	Pastarätter	Pasta dishes	1
5521	Pastagratäng	Pasta gratin	Pastarätter	Pasta dishes	1
2696	Pastagratäng med ostsås och skinka	Pasta au gratin w/ cheese sauce and ham	Pastarätter	Pasta dishes	1
861	Ravioli m sås fryst el konserv	Pasta ravioli w/ sauce frozen or canned	Pastarätter	Pasta dishes	1
853	Stuvade makaroner	Pasta macaroni in thick white sauce	Pastarätter	Pasta dishes	1
862	Tortellini m köttfyllning	Tortellini w/ meat filling	Pastarätter	Pasta dishes	1
518	Wokgrönsaker m kyckling nudlar	Stir fry vegetables w/ chicken noodles	Pastarätter	Pasta dishes	1
543	Wokgrönsaker m nudlar	Stir fry vegetables w/ noodles	Pastarätter	Pasta dishes	1
5572	Bagel med pålägg	Bagel w/ spread	Pizza paj pirog färdig smörgås	Pizza pie pirog	1
5579	Baguette sub med pålägg	Baguette sub w/ spread	Pizza paj pirog färdig smörgås	Pizza pie pirog	1
744	Ciabatta med mozzarella soltorkad tomat sallad	Ciabatta sandwich w/ mozzarella cheese sundried tomatoes and green salad	Pizza paj pirog färdig smörgås	Pizza pie pirog	1
5931	Enchiladas	Enchiladas	Pizza paj pirog färdig smörgås	Pizza pie pirog	1
5576	Focaccia med pålägg	Foccacia w/ spread	Pizza paj pirog färdig smörgås	Pizza pie pirog	1
5573	Fralla med pålägg	French roll w/ spread	Pizza paj pirog färdig smörgås	Pizza pie pirog	1
5575	Grillad macka med pålägg	Grilled sandwich w/ spread	Pizza paj pirog färdig smörgås	Pizza pie pirog	1
3011	Grönsakspaj	Vegetable pie	Pizza paj pirog färdig smörgås	Pizza pie pirog	1
2506	Kebab m bröd sallad sås	Döner kebab	Pizza paj pirog färdig smörgås	Pizza pie pirog	1
2062	Knäckemacka vete olika fyllningar typ Wasa Sandwich	Wheat crisp bread sandwich average diff. fillings	Pizza paj pirog färdig smörgås	Pizza pie pirog	1

[a] Foods in validation study and main survey Riksmaten ungdom 2016-17 = 1; Foods in main survey, only = 2; Foods in validation study, only = 3

Number	Food item (Swedish)	Food item	Main food group (Swedish)	Main food group	Study[a]
793	Matmuffins grov fullkorn m getost fårost broccoli spenat soltorkade tomater	Savory muffin coarse wholemeal flour w/ goat's or sheep's cheese broccoli spinach and sundried tomatoes	Pizza paj pirog färdig smörgås	Pizza pie pirog	1
3009	Ostpaj	Cheese pie	Pizza paj pirog färdig smörgås	Pizza pie pirog	1
757	Paj kyckling bacon	Chicken pie	Pizza paj pirog färdig smörgås	Pizza pie pirog	1
756	Paj köttfärs	Meat pie	Pizza paj pirog färdig smörgås	Pizza pie pirog	1
796	Paj ost skinka	Cheese and ham pie	Pizza paj pirog färdig smörgås	Pizza pie pirog	1
758	Pirog m köttfärsfyllning	Pirogue filled w/ minced meat	Pizza paj pirog färdig smörgås	Pizza pie pirog	1
3683	Pirog vegetarisk quorn el soja	Vegetarian pirogue	Pizza paj pirog färdig smörgås	Pizza pie pirog	1
788	Pitabröd m falafel sallad sesam pepperoni	Pitta bread w/ falafel green salad sesame and pepperoni	Pizza paj pirog färdig smörgås	Pizza pie pirog	1
5912	Pizza hemlagad	Pizza homemade	Pizza paj pirog färdig smörgås	Pizza pie pirog	1
5913	Pizza kyl/frysvara	Pizza frozen or chilled	Pizza paj pirog färdig smörgås	Pizza pie pirog	1
799	Pizza ospec.	Pizza unspec.	Pizza paj pirog färdig smörgås	Pizza pie pirog	1
765	Pizza pan pizza rökt griskött fryst värmd	Pizza pan pizza smoked pork frozen heated	Pizza paj pirog färdig smörgås	Pizza pie pirog	1
3063	Pizzabullar	Mini pizzas w/ ham cheese tomatoes and/or other fillings	Pizza paj pirog färdig smörgås	Pizza pie pirog	1
5578	Sandwich med pålägg	Sandwich w/ spread	Pizza paj pirog färdig smörgås	Pizza pie pirog	1
771	Skaldjurspaj fryst värmd	Shellfish pie frozen heated	Pizza paj pirog färdig smörgås	Pizza pie pirog	1
4212	Smördegs- eller filodegsinbakad ost	Puff pastry or filo pastry baked cheese	Pizza paj pirog färdig smörgås	Pizza pie pirog	1
5814	Smörgås subway	Subway sandwich	Pizza paj pirog färdig smörgås	Pizza pie pirog	1
797	Smörgåstårta landgång	Savory sandwich layer-cake filled w/ liver paté cottage cheese ham	Pizza paj pirog färdig smörgås	Pizza pie pirog	1
5574	Toast med pålägg	Toast w/ spread	Pizza paj pirog färdig smörgås	Pizza pie pirog	1
3680	Vegetarisk paj med quornfärs	Vegetarian pie w/ quorn	Pizza paj pirog färdig smörgås	Pizza pie pirog	1
5577	Wrap med pålägg	Wrap w/ spread	Pizza paj pirog färdig smörgås	Pizza pie pirog	1
750	Vårrulle fryst värmd	Spring roll frozen heated	Pizza paj pirog färdig smörgås	Pizza pie pirog	1
4511	Färskpotatis kokt m salt	New potatoe boiled w/ salt	Potatis	Potatoes	1
231	Höstpotatis kokt m salt	Old potato boiled w/ salt	Potatis	Potatoes	1
238	Klyftpotatis u skal ugnsstekt m rapsolja	Potato wedges w/ skin oven-baked in rapeseed oil	Potatis	Potatoes	1
233	Potatis m skal bakad u salt	Potato w/ skins unsalted baked	Potatis	Potatoes	1
2652	Potatis pressad	Pressed potatoes	Potatis	Potatoes	1
5732	Potatis stekt och råstekt	Potato fried	Potatis	Potatoes	1
4128	Hasselbackspotatis bakad	Potatoes ovenbaked w/ butter, salt and breadcrumbs	Potatisprodukter potatisrätter	Potatoes products and dishes	1
257	Janssons frestelse	Swedish potato gratin w/ pickled sprats coffee cream and milk Jansson's temptation	Potatisprodukter potatisrätter	Potatoes products and dishes	1

[a] Foods in validation study and main survey Riksmaten ungdom 2016-17 = 1; Foods in main survey, only = 2; Foods in validation study, only = 3

Number	Food item (Swedish)	Food item	Main food group (Swedish)	Main food group	Study[a]
251	Kroppkakor o pitepalt	Potato dumplings stuffed w/ chopped pork	Potatisprodukter potatisrätter	Potatoes products and dishes	1
5499	Kycklingpytt	Swedish chicken hash	Potatisprodukter potatisrätter	Potatoes products and dishes	1
5731	Pommes frites	French fries	Potatisprodukter potatisrätter	Potatoes products and dishes	1
273	Potatisbullar stekta	Potato patties	Potatisprodukter potatisrätter	Potatoes products and dishes	1
2891	Potatisgnocchi	Potato gnocchi	Potatisprodukter potatisrätter	Potatoes products and dishes	1
284	Potatisgratäng grädde ost fryst värmd	Potatoes au gratin w/ cream and cheese frozen heated	Potatisprodukter potatisrätter	Potatoes products and dishes	1
4209	Potatisgratäng med skinka eller annat kött	Potatoes au gratin w/ ham or other meat	Potatisprodukter potatisrätter	Potatoes products and dishes	1
282	Potatiskroketter frysta värmda	Potato croquettes frozen heated	Potatisprodukter potatisrätter	Potatoes products and dishes	1
274	Potatismos	Mashed potatoes	Potatisprodukter potatisrätter	Potatoes products and dishes	1
283	Potatismos pulver berik tillagad	Mashed potatoes instant powder fortified prepared	Potatisprodukter potatisrätter	Potatoes products and dishes	1
261	Potatissallad gräddfil majonnäs	Potato salad w/ sour cream mayonnaise	Potatisprodukter potatisrätter	Potatoes products and dishes	1
264	Potatissallad vinägrettsås	Potato salad w/ vinaigrette	Potatisprodukter potatisrätter	Potatoes products and dishes	1
272	Potatisstuvning	Potatoes creamed	Potatisprodukter potatisrätter	Potatoes products and dishes	1
249	Pytt i panna gjutjärnsstekpanna hemlagad	Swedish hash in cast iron frying pan home-made	Potatisprodukter potatisrätter	Potatoes products and dishes	1
263	Pytt i panna vegetarisk rotfrukt svamp	Potatoes and vegetables fried	Potatisprodukter potatisrätter	Potatoes products and dishes	1
271	Raggmunkar potatisplättar potatispannkakor hemlagade	Potato pancakes home-made	Potatisprodukter potatisrätter	Potatoes products and dishes	1
281	Schweizisk potatiskaka rösti fryst värmd	Swiss potato cake rösti frozen heated	Potatisprodukter potatisrätter	Potatoes products and dishes	1
2125	Glasnudlar kokta m salt	Cellophane noodles boiled w/ salt	Ris risnudlar	Rice rice noodles	1
820	Ris olika sorter kokt m salt	Rice diff. kinds boiled w/ salt	Ris risnudlar	Rice rice noodles	1
818	Ris vildris kokt	Wild rice boiled	Ris risnudlar	Rice rice noodles	1
2517	Råris fullkorn långkornigt kokt m salt	Long-grain brown rice boiled	Ris risnudlar	Rice rice noodles	1
5563	Riskaka salt och smaksatt	Rice cake	Riskakor	Rice cakes	1
842	Paella	Paella	Risrätter	Rice diches	1

[a] Foods in validation study and main survey Riksmaten ungdom 2016-17 = 1; Foods in main survey, only = 2; Foods in validation study, only = 3

Number	Food item (Swedish)	Food item	Main food group (Swedish)	Main food group	Study[a]
2686	Ris kokt med fräst lök och buljong, pilaffris	Rice w/ onion pilaff rice	Risrätter	Rice diches	1
2683	Ris persiskt	Rice persian	Risrätter	Rice diches	1
2687	Risotto italiensk (med parmesanost)	Italian risotto w/ parmesan cheese	Risrätter	Rice diches	1
2685	Rispytt med kött kyckling eller korv	Fried rice w/ meat chicken or sausage	Risrätter	Rice diches	1
5802	Kaviar på tub	Fish spawn paste spread smoked	Rom, kaviar	Roe, roe spread	1
5801	Rom fiskrom	Fish spawn	Rom, kaviar	Roe, roe spread	1
2791	Gari (inlagd japansk ingefära)	Ginger pickles	Rotfrukter	Root vegetables	1
3569	Grönsaksblandning morötter palsternacka purjolök rotselleri kokt	Mixed vegetables carrot parsnip leek celeriac boiled	Rotfrukter	Root vegetables	1
288	Kålrot	Swede	Rotfrukter	Root vegetables	1
3899	Kålrot stekt eller wokad	Swede fried or woked	Rotfrukter	Root vegetables	1
289	Morot	Carrot	Rotfrukter	Root vegetables	1
2608	Morot stekt eller wokad	Carrot pan-fried or stir-fried	Rotfrukter	Root vegetables	1
291	Pepparrot	Horseradish	Rotfrukter	Root vegetables	1
2613	Rotselleri stekt eller wokad	Celeriac pan-fried or stir-fried	Rotfrukter	Root vegetables	1
293	Rädisa	Radish	Rotfrukter	Root vegetables	1
294	Rödbeta	Beetroot	Rotfrukter	Root vegetables	1
319	Rödbeta inlagd u lag	Beetroots pickled drained	Rotfrukter	Root vegetables	1
2614	Rödbeta stekt eller wokad	Beetroot pan-fried or stir-fried	Rotfrukter	Root vegetables	1
3771	Sötpotatis ugnsstekt	Sweet potato fried	Rotfrukter	Root vegetables	1
2672	Ugnsrostade grönsaker och rotfrukter	Vegetables and root vegetables baked	Rotfrukter	Root vegetables	1
4025	Wasabi	Wasabi	Rotfrukter	Root vegetables	1
3523	Ciderkaraktär alkoholfri	Cider non-alcoholic	Saft läsk cider u alkohol	Fruit syrup soft drink, non-alcoholic cider	1
1829	Coladryck	Cola drink	Saft läsk cider u alkohol	Fruit syrup soft drink, non-alcoholic cider	1
3526	Fruktdryck utan kolsyra typ Festis	Fruit syrup soft drink non-carbonated	Saft läsk cider u alkohol	Fruit syrup soft drink, non-alcoholic cider	1
2303	Ice tea drickf	Ice tea RTD	Saft läsk cider u alkohol	Fruit syrup soft drink, non-alcoholic cider	1
1828	Läsk m kolsyra	Soft drinks carbonated	Saft läsk cider u alkohol	Fruit syrup soft drink, non-alcoholic cider	1
1839	Måltidsdricka äpple lingon drickf berik	Apple cowberry drink RTD fortified	Saft läsk cider u alkohol	Fruit syrup soft drink, non-alcoholic cider	1
1818	Måltidsdryck drickf	Cordial reduced sugar RTD	Saft läsk cider u alkohol	Fruit syrup soft drink, non-alcoholic cider	1
669	Nektar drickf	Nectars RTD	Saft läsk cider u alkohol	Fruit syrup soft drink, non-alcoholic cider	1
1820	Saft drickf	Fruit syrup RTD	Saft läsk cider u alkohol	Fruit syrup soft drink, non-alcoholic cider	1

[a] Foods in validation study and main survey Riksmaten ungdom 2016-17 = 1; Foods in main survey, only = 2; Foods in validation study, only = 3

Number	Food item (Swedish)	Food item	Main food group (Swedish)	Main food group	Study[a]
3733	Saftglögg	Nectar w/ spices "gluhwein"	Saft läsk cider u alkohol	Fruit syrup soft drink, non-alcoholic cider	1
5184	Tranbärsdryck drickf	Cranberry drink RTD	Saft läsk cider u alkohol	Fruit syrup soft drink, non-alcoholic cider	1
5716	Bulgursallad falafel	Salad w/ bulgur and falafel	Sallad blandad mat	Mixed salad	1
3099	Bön- och kikärtssallad	Salad w/ beans chickpeas	Sallad blandad mat	Mixed salad	1
513	Caesarsallad kycklingsallad m dressing	Chicken salad w/ dressing	Sallad blandad mat	Mixed salad	1
529	Grekisk sallad	Greek sallad	Sallad blandad mat	Mixed salad	1
3093	Kycklingsallad	Chicken salad	Sallad blandad mat	Mixed salad	1
3095	Laxsallad	Salad w/ salmon or other fish	Sallad blandad mat	Mixed salad	1
3097	Ost- och skinksallad	Cheese and ham salad	Sallad blandad mat	Mixed salad	1
4093	Pastasallad med bönor kikärtor	Pasta salad w/ beans chickpeas	Sallad blandad mat	Mixed salad	1
3092	Pastasallad med kyckling	Pasta salad w/ chicken	Sallad blandad mat	Mixed salad	1
3773	Pastasallad med lax	Pasta salad w/ salmon or other fish	Sallad blandad mat	Mixed salad	1
3103	Pastasallad med ost och skinka	Pasta salad w/ cheese and ham	Sallad blandad mat	Mixed salad	1
3104	Pastasallad med rostbiff	Pasta salad w/ roast beef or other meat	Sallad blandad mat	Mixed salad	1
3110	Pastasallad med räkor	Pasta salad w/ shrimps or other types of shellfish	Sallad blandad mat	Mixed salad	1
3774	Pastasallad med tonfisk	Pasta salad w/ tuna	Sallad blandad mat	Mixed salad	1
3098	Rostbiffssallad	Roast beef salad	Sallad blandad mat	Mixed salad	1
3094	Räksallad	Shellfish salad shrimp salad	Sallad blandad mat	Mixed salad	1
5717	Sallad med rödbetor och chèvre	Salad w/ chevré	Sallad blandad mat	Mixed salad	1
3060	Tabouleh	Tabbouleh	Sallad blandad mat	Mixed salad	1
5927	Tacosallad	Taco salad	Sallad blandad mat	Mixed salad	1
3096	Tonfisksallad	Tuna salad	Sallad blandad mat	Mixed salad	1
1968	Chilisås tomat	Tomato chili sauce	Senap ketchup HP-sås soja "smaksättare"	Mustard ketchup HP sauce soja "flavouring"	1
5908	Dip till kyckling nuggets	Dip for chicken nuggets	Senap ketchup HP-sås soja "smaksättare"	Mustard ketchup HP sauce soja "flavouring"	1
1970	HP-sås	HP-sauce	Senap ketchup HP-sås soja "smaksättare"	Mustard ketchup HP sauce soja "flavouring"	1
1969	Ketchup	Tomato ketchup	Senap ketchup HP-sås soja "smaksättare"	Mustard ketchup HP sauce soja "flavouring"	1
1972	Senap svensk	Mustard Swedish	Senap ketchup HP-sås soja "smaksättare"	Mustard ketchup HP sauce soja "flavouring"	1
909	Sojasås	Soy sauce	Senap ketchup HP-sås soja "smaksättare"	Mustard ketchup HP sauce soja "flavouring"	1
2007	Sweet chilisås	Sweet chili sauce	Senap ketchup HP-sås soja "smaksättare"	Mustard ketchup HP sauce soja "flavouring"	1

[a] Foods in validation study and main survey Riksmaten ungdom 2016-17 = 1; Foods in main survey, only = 2; Foods in validation study, only = 3

Number	Food item (Swedish)	Food item	Main food group (Swedish)	Main food group	Study[a]
2012	Teriyakisås	Teryaki sauce Japanese	Senap ketchup HP-sås soja "smaksättare"	Mustard ketchup HP sauce soja "flavouring"	1
1395	Räkor	Shrimps raw	Skaldjur bläckfisk färsk fryst kokt	Shellfish squid fresh frozen cooked	1
5779	Mjukost	Processed cheese	Smältost	Processed cheese	1
3864	Kryddsmör	Butter flavoured w/ garlic or parsley	Smör	Butter	1
29	Smör fett 80 %	Butter 80 % fat	Smör	Butter	1
5564	Kex smörgåskex litet	Cracker small	Smörgåskex	Salty biscuits	1
5565	Kex smörgåskex stort	Cracker semi-sweet biscuit big	Smörgåskex	Salty biscuits	1
1633	Smörgåsrån	Plain wafers	Smörgåskex	Salty biscuits	1
1897	Druvsocker	Glucose	Socker sirap honung	Sugar syrup honey	1
1896	Honung	Honey	Socker sirap honung	Sugar syrup honey	1
1894	Ljus sirap	Golden syrup	Socker sirap honung	Sugar syrup honey	1
1892	Socker	Sugar	Socker sirap honung	Sugar syrup honey	1
1883	Tabletter sockerfria	Pastilles sugar free	Sockerfritt godis	Sweets sugar-free	1
2066	Sojakorv	Soya sausage	Sojaprotein veteprotein Quorn produkter och rätter	Soy and wheat protein quorn products and dishes	1
544	Vegetabilisk pastej typ tartex	Vegetable paste Tartex	Sojaprotein veteprotein Quorn produkter och rätter	Soy and wheat protein quorn products and dishes	1
5714	Vegetarisk biff	Vegetarian beef	Sojaprotein veteprotein Quorn produkter och rätter	Soy and wheat protein quorn products and dishes	1
5709	Vegetarisk filé bitar	Vegetarian filet	Sojaprotein veteprotein Quorn produkter och rätter	Soy and wheat protein quorn products and dishes	1
5715	Vegetarisk färs	Vegetarian minced quorn soy	Sojaprotein veteprotein Quorn produkter och rätter	Soy and wheat protein quorn products and dishes	1
5712	Vegetarisk färssås	Vegetarian bolognese sauce	Sojaprotein veteprotein Quorn produkter och rätter	Soy and wheat protein quorn products and dishes	1
5713	Vegetarisk gryta, quorn eller soja	Vegetarian stew	Sojaprotein veteprotein Quorn produkter och rätter	Soy and wheat protein quorn products and dishes	1
5703	Vegetarisk lasagne	Vegetarian lasagne	Sojaprotein veteprotein Quorn produkter och rätter	Soy and wheat protein quorn products and dishes	1
5704	Vegetarisk moussaka	Vegetarian moussaka	Sojaprotein veteprotein Quorn produkter och rätter	Soy and wheat protein quorn products and dishes	1
5706	Vegetarisk pyttipanna	Vegetarian swedish hash	Sojaprotein veteprotein Quorn produkter och rätter	Soy and wheat protein quorn products and dishes	1
5705	Vegetarisk schnitzel	Vegetarian schnitzel	Sojaprotein veteprotein Quorn produkter och rätter	Soy and wheat protein quorn products and dishes	1
5711	Vegetariska bollar	Vegetarian balls	Sojaprotein veteprotein Quorn produkter och rätter	Soy and wheat protein quorn products and dishes	1

[a] Foods in validation study and main survey Riksmaten ungdom 2016-17 = 1; Foods in main survey, only = 2; Foods in validation study, only = 3

Number	Food item (Swedish)	Food item	Main food group (Swedish)	Main food group	Study[a]
5710	Vegetariska nuggets	Vegetarian nuggets	Sojaprotein veteprotein Quorn produkter och rätter	Soy and wheat protein quorn products and dishes	1
1354	Bouillabaisse fisksoppa	Fish soup	Soppa mat	Soup	1
438	Grönsakssoppa redd ängamat	Vegetable soup w/ cream	Soppa mat	Soup	1
1209	Kycklingsoppa redd pulver tillagad m vatten typ Varma koppen	Instant chicken soup cream of cooked w/ water e.g. Varma koppen	Soppa mat	Soup	1
1206	Kycklingsoppa thailändsk	Chicken soup clear	Soppa mat	Soup	1
1073	Köttsoppa nötkött	Beef soup	Soppa mat	Soup	1
903	Linssoppa	Lentil soup	Soppa mat	Soup	1
2992	Misosoppa	Miso soup w/ tofu	Soppa mat	Soup	1
873	Nudelsoppa olika smaker kryddad	Instant noodles soup diff. flavours	Soppa mat	Soup	1
900	Ärtsoppa m fläsk konserv tillagad m vatten	Pea soup w/ pork canned prepared w/ water	Soppa mat	Soup	1
904	Ärtsoppa vegetarisk	Pea soup veg.	Soppa mat	Soup	1
2302	Energidryck typ Red Bull berikad	Energy drink e.g. Red Bull fortified	Sportdrycker energidrycker	Sports drinks, energy drinks	1
1918	Brännvin renat el vodka vol. % 40	Vodka or snaps vol. % 40	Starksprit	Spirits	1
333	Champinjoner färska	Mushrooms	Svamp	Mushroom	1
457	Champinjoner stekta	Mushrooms fried	Svamp	Mushroom	1
2619	Kantareller stekta	Chanterelles fried	Svamp	Mushroom	1
3317	Svamp stekt	Mushroom fried	Svamp	Mushroom	1
3557	Chutney	Chutney	Sylt marmelad gelé äppelmos o dyl	Jam, marmelade, jelly, apple sauce	1
1812	Cumberlandsås	Cumberland sauce	Sylt marmelad gelé äppelmos o dyl	Jam, marmelade, jelly, apple sauce	1
1798	Lingonsylt	Cowberry/lingonberry jam	Sylt marmelad gelé äppelmos o dyl	Jam, marmelade, jelly, apple sauce	1
5552	Marmelad	Jam orange, apricot and rasberry	Sylt marmelad gelé äppelmos o dyl	Jam, marmelade, jelly, apple sauce	1
1795	Svartvinbärsgelé	Black currant jelly	Sylt marmelad gelé äppelmos o dyl	Jam, marmelade, jelly, apple sauce	1
5548	Sylt frukt och bär	Jam, strawberry, rasberry and blueberry	Sylt marmelad gelé äppelmos o dyl	Jam, marmelade, jelly, apple sauce	1
1809	Äppelmos	Apple sauce	Sylt marmelad gelé äppelmos o dyl	Jam, marmelade, jelly, apple sauce	1
1553	Kalvsylta	Veal brawn	Sylta	Brawn	1
3598	Barbequesås	Barbeque sauce	Sås dressing majonnäs	Sauce dressing mayonnaise	1
58	Bearnaisesås	Béarnaise sauce	Sås dressing majonnäs	Sauce dressing mayonnaise	1
1985	Béchamelsås	White sauce béchamel	Sås dressing majonnäs	Sauce dressing mayonnaise	1
1991	Brunsås mjölk	Brown sauce w/ milk	Sås dressing majonnäs	Sauce dressing mayonnaise	1

[a] Foods in validation study and main survey Riksmaten ungdom 2016-17 = 1; Foods in main survey, only = 2; Foods in validation study, only = 3

Number	Food item (Swedish)	Food item	Main food group (Swedish)	Main food group	Study[a]
3869	Caesardressing	Ceasar dressing	Sås dressing majonnäs	Sauce dressing mayonnaise	1
2104	Carbonara sås	Carbonara	Sås dressing majonnäs	Sauce dressing mayonnaise	1
459	Champinjonsås	Mushrooms in white sauce	Sås dressing majonnäs	Sauce dressing mayonnaise	1
2075	Coleslaw kall sås	Cold sauce school coleslaw	Sås dressing majonnäs	Sauce dressing mayonnaise	1
5906	Dip till pommes frites bearnaise	Dip for french fries bearnaise	Sås dressing majonnäs	Sauce dressing mayonnaise	1
23	Gravlaxsås hovmästarsås	Sauce (mustard based) for raw spiced salmon	Sås dressing majonnäs	Sauce dressing mayonnaise	1
2973	Hamburgerdressing	Hamburger dressing	Sås dressing majonnäs	Sauce dressing mayonnaise	1
2073	Jordnötssås	Peanut sauce	Sås dressing majonnäs	Sauce dressing mayonnaise	1
21	Majonnäs fett 90%	Mayonnaise 90% fat	Sås dressing majonnäs	Sauce dressing mayonnaise	1
2142	Ost- och skinksås	Cheese and ham sauce	Sås dressing majonnäs	Sauce dressing mayonnaise	1
1989	Ostsås ädelost	Blue cheese sauce made w/ coffee cream and whipping cream	Sås dressing majonnäs	Sauce dressing mayonnaise	1
3868	Pepparrotsvisp	Whipped cream w/ horseraddish	Sås dressing majonnäs	Sauce dressing mayonnaise	1
2004	Pestosås m basilika pinjenötter cashewnötter	Pesto sauce w/ basil pine nuts and cashew nuts	Sås dressing majonnäs	Sauce dressing mayonnaise	1
5911	Pizzatopping sås	Pizza topping sauce	Sås dressing majonnäs	Sauce dressing mayonnaise	1
25	Remouladsås	Rémoulade sauce	Sås dressing majonnäs	Sauce dressing mayonnaise	1
60	Rhode Island sås	Rhode Island sauce	Sås dressing majonnäs	Sauce dressing mayonnaise	1
2969	Skaldjurssås	Shellfish sauce	Sås dressing majonnäs	Sauce dressing mayonnaise	1
1992	Sky	Thin gravy	Sås dressing majonnäs	Sauce dressing mayonnaise	1
462	Tomatsalsa kall	Tomato sauce Mexican style	Sås dressing majonnäs	Sauce dressing mayonnaise	1
2000	Tomatsås	Tomato sauce	Sås dressing majonnäs	Sauce dressing mayonnaise	1
26	Vinägrett dressing fett 45%	Vinaigrette w/ water 45% fat	Sås dressing majonnäs	Sauce dressing mayonnaise	1
3586	Yoghurtsås	Yoghurt sauce	Sås dressing majonnäs	Sauce dressing mayonnaise	1
2002	Äggsås m persilja	Egg sauce w/ parsley	Sås dressing majonnäs	Sauce dressing mayonnaise	1
1756	Bär- fruktkräm bär- fruktsoppa sötad m sorbitol ospec	Soup fruit berries sweetener unspec.	Söta soppor kräm o efterrättssås	Sweet soups sauce	1
2902	Bärsås	Berry sauce	Söta soppor kräm o efterrättssås	Sweet soups sauce	1
1768	Chokladsås	Chocolate sauce	Söta soppor kräm o efterrättssås	Sweet soups sauce	1
1749	Fruktdryck blåbär	Fruit drink blueberry	Söta soppor kräm o efterrättssås	Sweet soups sauce	1
1750	Fruktsoppa ätf blandad torkad frukt pastöriserad berik	Fruit soup	Söta soppor kräm o efterrättssås	Sweet soups sauce	1
1769	Kolasås	Butterscotch sauce	Söta soppor kräm o efterrättssås	Sweet soups sauce	1
1744	Kräm bär frukt ospec.	Stewed fruit and berries	Söta soppor kräm o efterrättssås	Sweet soups sauce	1
1751	Nyponsoppa ätf pastöriserad el på pulver berik	Rosehip soup RTE pasteurised or powder prepared fortified	Söta soppor kräm o efterrättssås	Sweet soups sauce	1
1748	Saftsås	Fruit syrup sauce thickened w/ potato starch	Söta soppor kräm o efterrättssås	Sweet soups sauce	1
1771	Vaniljsås m grädde mjölk	Custard	Söta soppor kräm o efterrättssås	Sweet soups sauce	1

[a] Foods in validation study and main survey Riksmaten ungdom 2016-17 = 1; Foods in main survey, only = 2; Foods in validation study, only = 3

Number	Food item (Swedish)	Food item	Main food group (Swedish)	Main food group	Study[a]
3112	Sötningsmedel (till kaffe, te m.m.)	Artificial sweetener	Sötningsmedel	Artificial sweetener	1
2557	Tacoskal	Taco shells	Tacoskal	Taco shell	1
1962	Te bryggt	Tea brewed	Te	Tea infusion	1
5602	Tuggummi ospec	Chewing gum unspec.	Tuggummi	Chewing gum	1
1953	Kranvatten	Tap water	Vatten mineralvatten	Water, mineral water	1
1954	Källvatten förpackat	Spring water packed	Vatten mineralvatten	Water, mineral water	1
1951	Mineralvatten	Mineral water	Vatten mineralvatten	Water, mineral water	1
5684	Havredryck sojadryck	Oat milk soy milk	Vegetabiliska produkter och mjölkersättning	Vegetable products and milk replacement	1
5690	Havregurt Soygurt	Oatgurt Soygurt milkfree yoghurt	Vegetabiliska produkter och mjölkersättning	Vegetable products and milk replacement	1
3732	Lättglögg 2,25%	Mulled wine (non-alcoholic)	Vin	Wine	1
4060	Mousserande vin, champagne	Sparkling wine champagne	Vin	Wine	1
1907	Rödvin vol. % 14	Red wine vol. % 14	Vin	Wine	1
1916	Sherry halvtorr vol. % 17	Sherry medium dry vol. % 17	Vin	Wine	1
1917	Starkvinsglögg vol. % 16	Fortified wine mulled vol. % 16	Vin	Wine	1
1912	Vinglögg vol. % 10	Wine mulled vol. % 10	Vin	Wine	1
1904	Vitt vin vol. % 1	White wine vol. % 1	Vin	Wine	1
1908	Vitt vin vol. % 12	White wine vol. % 12	Vin	Wine	1
694	Välling för vuxna fullkorn berik	Gruel for adults wholemeal fortified	Välling	Gruel	1
2205	Ägg konventionellt kokt	Boiled egg conventional	Ägg	Egg	1
1225	Ägg konventionellt rått	Egg raw conventional	Ägg	Egg	1
1233	Ägg konventionellt stekt	Egg fried conventional	Ägg	Egg	1
1199	Bondomelett	Farmer's omelette	Äggprodukter o rätter	Egg products and dishes	1
1230	Fransk omelett vatten	French omelette w/ water	Äggprodukter o rätter	Egg products and dishes	1
2197	Äggröra	Scrambled eggs w/o flour	Äggprodukter o rätter	Egg products and dishes	1
2305	Alkoholfri öl	Non alcoholic beer	Öl	Beer	1
1901	Öl lättöl vol. % 2,3	Small beer or low-alcohol beer vol. % 2.3	Öl	Beer	1
1902	Öl pilsner vol. % 3,5	Beer or pilsner vol. % 3.5	Öl	Beer	1
1906	Öl starköl el exportöl vol. % 5,4	Strong beer vol. % 5.4	Öl	Beer	1
3980	Dryck med vitaminer och mineraler typ Vitamin well	Water w/ vitamins and minerals added	Övriga sötade drycker vattenchoklad	Beverages miscellaneous	1
667	Multivitamindryck tillverkad av konc drickf	Multi-vitamin beverage prepared from concentrate RTD	Övriga sötade drycker vattenchoklad	Beverages miscellaneous	1
4	Kokosfett	Coconut butter	Övrigt fett (ister, talg, kokosfett)	Other fats (lard, tallow, coconut oil)	1
5996	Bönpasta	Pasta made of beans	Baljväxter (bönor, linser och ärter)	Leguminous plant	2
2947	Laxbullar laxburgare	Salmon fishballs, fish burger fried	Fisk o skaldjursprodukter o rätter	Fish and shellfish dishes	2
5993	Flingor med röda bär	Cereals w/ red fruit	Flingor - frukostflingor	Breakfast cereals	2
6013	Kycklinggryta i currysås	Chicken curry stew	Fågelprodukter fågelrätter	Poultry products and dishes	2

[a] Foods in validation study and main survey Riksmaten ungdom 2016-17 = 1; Foods in main survey, only = 2; Foods in validation study, only = 3

Number	Food item (Swedish)	Food item	Main food group (Swedish)	Main food group	Study[a]
2863	Kycklinggryta med grädde/crème fraiche	Chicken casserole w/ cream/crème fraiche	Fågelprodukter fågelrätter	Poultry products and dishes	2
1100	Kycklingköttbullar fett 12% stekta frysta	Chicken meat balls fat 12% fried frozen	Fågelprodukter fågelrätter	Poultry products and dishes	2
6042	Kokosvatten smaksatt el naturell	Coconut water natural or flavoured	Grönsaker	Vegetables excluding potatoes	2
495	Majskolv kokt	Corn cobs boiled	Grönsaker	Vegetables excluding potatoes	2
1886	Kakaopulver fett 20-22%	Cocoa powder fat 20-22%	Kakaoprodukter	Cacao produkts	2
1517	Salami importerad fett 45%	Salami imported fat 45%	Korv	Sausages	2
6015	Lammgryta med curry	Lamb curry	Köttprodukter kötträtter	Meat products meat dishes	2
114	Filmjölk fett 3%	Fermented milk fat 3%	Naturell fil yoghurt	Natural fermented products	2
120	Lättfil fett 0,5%	Fermented milk skimmed fat 0.5%	Naturell fil yoghurt	Natural fermented products	2
135	Lättyoghurt naturell fett 0,5%	Yoghurt natural skimmed fat 0.5%	Naturell fil yoghurt	Natural fermented products	2
130	Mellanfil fett 1,5%	Fermented milk semi-skimmed fat 1.5%	Naturell fil yoghurt	Natural fermented products	2
124	Yoghurt naturell fett 3 %	Yoghurt natural fat 3%	Naturell fil yoghurt	Natural fermented products	2
144	Filmjölk m jordgubbssmak fett 2,7%	Fermented milk strawberry flavour 2.7% fat	Smaksatt fil yoghurt	Flavoured fermented milk	2
145	Lättfil m jordgubbsmak 0,5% fett	Fermented milk skimmed strawberry flavour 0.5% fat	Smaksatt fil yoghurt	Flavoured fermented milk	2
6040	Yoghurt smaksatt helfet	Yoghurt flavoured full fat	Smaksatt fil yoghurt	Flavoured fermented milk	2
6041	Yoghurt smaksatt lätt	Yoghurt flavoured light	Smaksatt fil yoghurt	Flavoured fermented milk	2
5758	Aioli	Aioli mayonnaise	Sås dressing majonnäs	Sauce dressing mayonnaise	2
2759	Currysås kylvara	Curry sauce RTE refrigerated/frozen	Sås dressing majonnäs	Sauce dressing mayonnaise	2
2230	Ostsås fett 8%	Cheese sauce ready-made sauce fat 8%	Sås dressing majonnäs	Sauce dressing mayonnaise	2
2144	Tzatziki	Tzatziki	Sås dressing majonnäs	Sauce dressing mayonnaise	2
5994	Vitlökssås	Garlic sauce	Sås dressing majonnäs	Sauce dressing mayonnaise	2
5520	Kycklinggryta med mango	Chicken stew w/ mango	Fågelprodukter fågelrätter	Poultry products and dishes	3
404	Sparris vit konserv u lag	Asparagus white canned drained	Grönsaker	Vegetables excluding potatoes	3
1847	Chokladdryck Pucko	Cocoa milk RTD Pucko	Mjölkdryck chokladdryck milkshake smothie m yoghurt	Cocoa milkdrink milkshake smothie w youghurt	3
5685	Filmjölk naturell	Fermented milk plain	Naturell fil yoghurt	Natural fermented products	3

[a] Foods in validation study and main survey Riksmaten ungdom 2016-17 = 1; Foods in main survey, only = 2; Foods in validation study, only = 3

Number	Food item (Swedish)	Food item	Main food group (Swedish)	Main food group	Study[a]
5687	Yoghurt naturell	Yoghurt plain	Naturell fil yoghurt	Natural fermented products	3
89	Margarinost fett 30%	Margarine cheese fat 30%	Ost med vegetabiliskt fett	Cheese with vegetable fat	3
5686	Filmjölk smaksatt	Fermented milk flavoured	Smaksatt fil yoghurt	Flavoured fermented milk	3
5688	Yoghurt smaksatt	Yoghurt flavoured	Smaksatt fil yoghurt	Flavoured fermented milk	3

[a] Foods in validation study and main survey Riksmaten ungdom 2016-17 = 1; Foods in main survey, only = 2; Foods in validation study, only = 3
