# Supplementary material for: Use of a Web-Based Dietary Assessment Tool (RiksmatenFlex) in Swedish Adolescents: Comparison and Validation Study
Source: J Med Internet Res. 2019 Oct 4;21(10):e12572. doi: 10.2196/12572 (PMC6914230; doi:10.2196/12572)
Supplement: Multimedia Appendix 3 [file jmir_v21i10e12572_app3.pdf]

Multimedia Appendix 3. Bland-Altman plots of reported intake of energy and macronutrients between the two dietary methods.

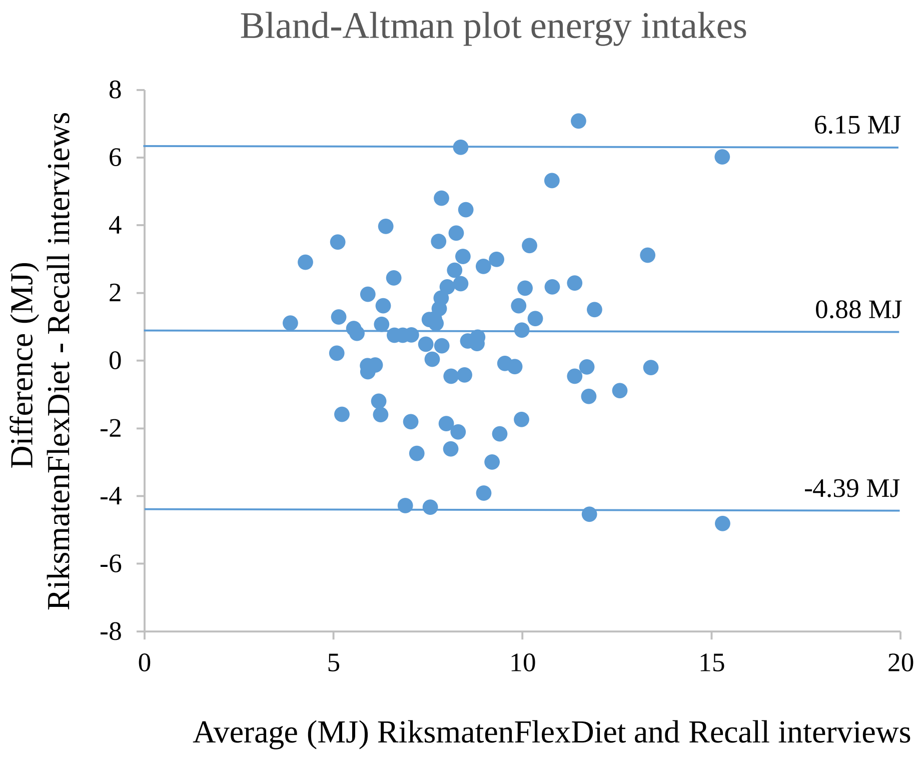

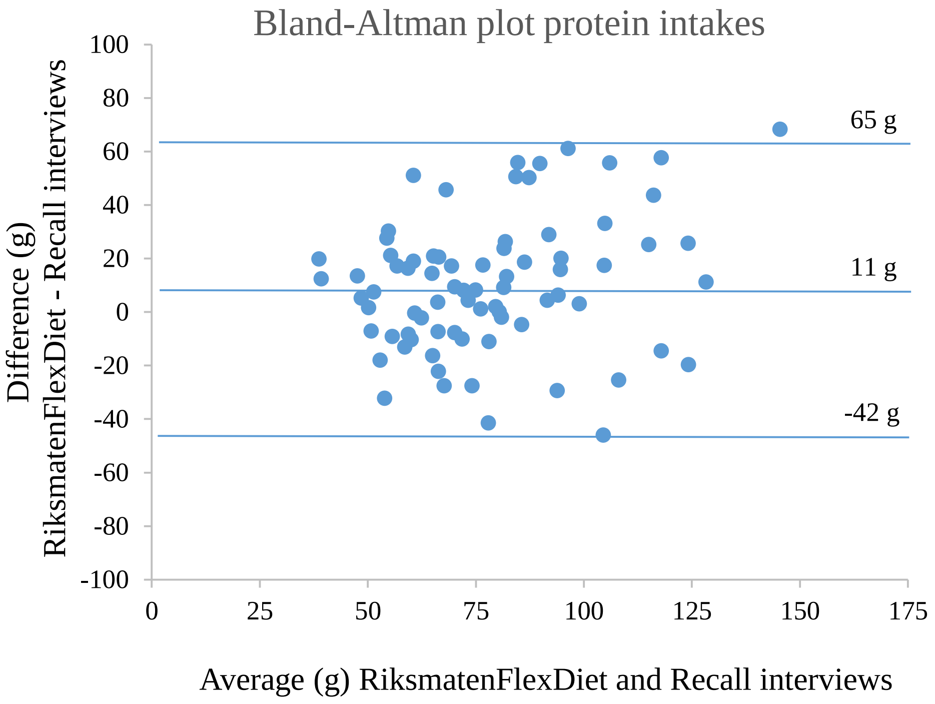

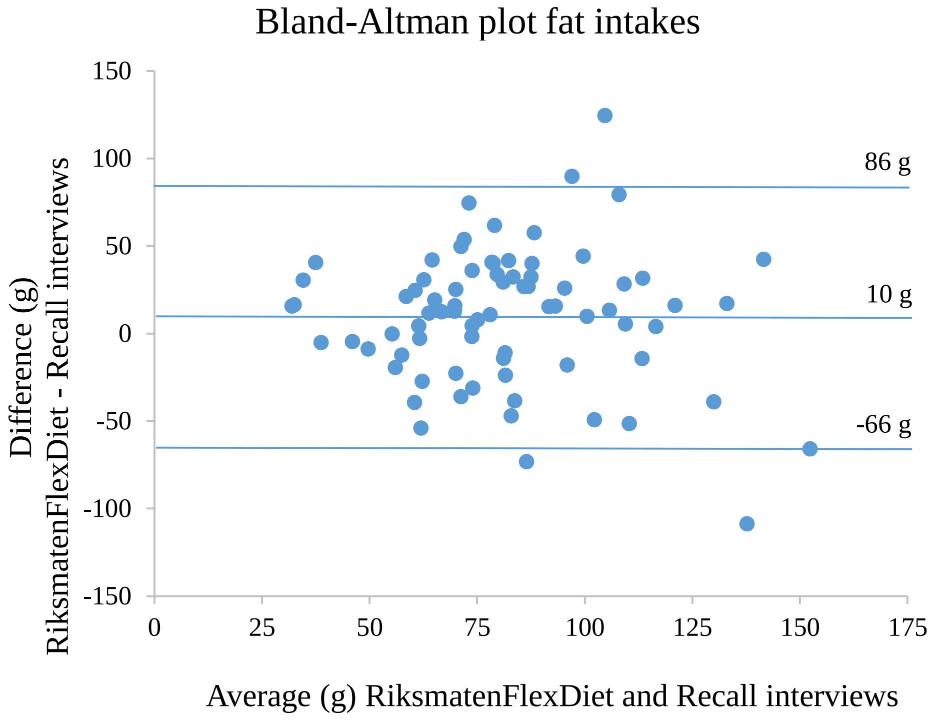
Bland-Altman plot fat intakes
Difference (g) RiksmatenFlexDiet - Recall interviews
Average (g) RiksmatenFlexDiet and Recall interviews
86 g
10 g
-66 g
150
100
50
0
-50
-100
-150
0
25
50
75
100
125
150
175

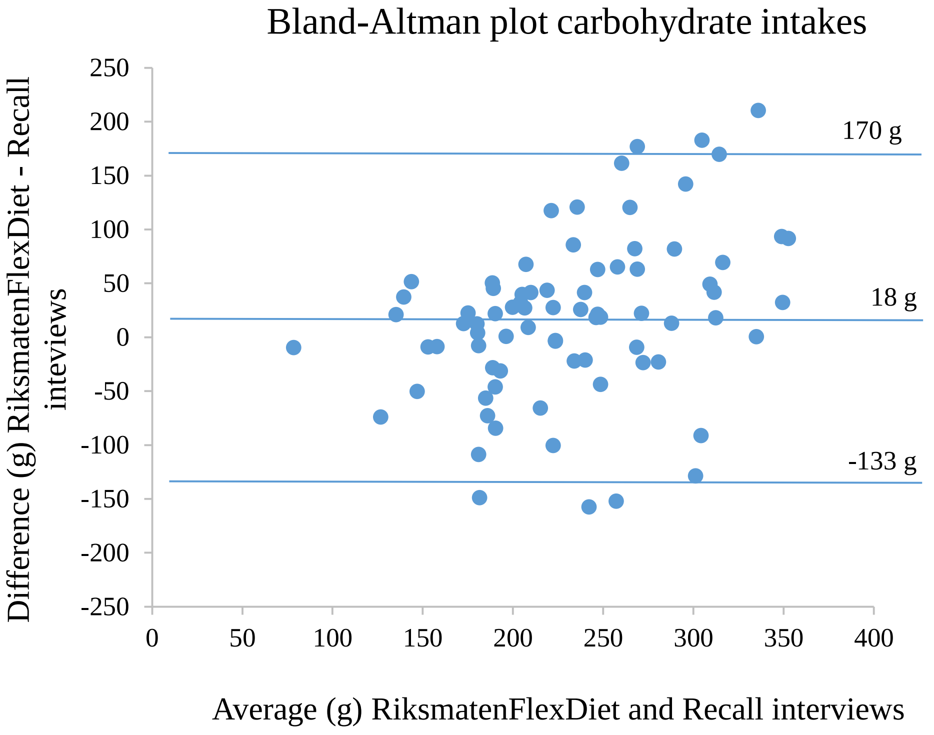
Bland-Altman plot carbohydrate intakes
Difference (g) RiksmatenFlexDiet - Recall inteviews
Average (g) RiksmatenFlexDiet and Recall interviews
170 g
18 g
-133 g
250
200
150
100
50
0
-50
-100
-150
-200
-250
0
50
100
150
200
250
300
350
400
